# Exciton-Tunable Phase Control and Superstrong Coupling through Multimode Polariton Engineering in Planar Waveguides

**DOI:** 10.1021/acsphotonics.6c01318

**Published:** 2026-10-16

**Authors:** Johannes Bürger, Erika Cortese, Simone Di Muzio, Daniele Sanvitto, Simone De Liberato, Dario Ballarini

**Affiliations:** † Institute of Nanotechnology, 518742 CNR Nanotec, Via Monteroni, Lecce 73100, Italy; ‡ School of Physics and Astronomy, University of Southampton, Southampton SO17 1BJ, U.K.; § Istituto di Fotonica e Nanotecnologie—Consiglio Nazionale delle Ricerche (CNR), Piazza Leonardo da Vinci 32, Milano 20133, Italy

**Keywords:** polaritonics, superstrong light-matter coupling, planar waveguides, integrated optics, ultrafast optical switching

## Abstract

Strong light–matter coupling in optical waveguides provides a versatile platform for engineering hybrid polaritonic modes and their dispersion. Here, we investigate multimode exciton–photon coupling in visible semiconductor waveguides supporting several transverse electric modes. Using rigorous coupled-wave analysis combined with a coupled-oscillator model, we show that the photonic band structure can be engineered across a range of regimes, from conventional multimode strong coupling to the superstrong coupling regime, where the Rabi coupling becomes comparable to the spacing between adjacent photonic modes. In the latter regime, hybridization between orthogonal electromagnetic modes is enabled by restricting the active material to a subregion of the mode volume where the photonic modes exhibit strong mutual overlap. This breaking of the orthogonality leads to polaritonic branches whose composition can be tuned among several photonic modes and the exciton. We demonstrate that small shifts of the exciton resonance produce pronounced changes in the propagation constants of different polariton branches, enabling exciton-controlled phase modulation through modal interference and, in the superstrong coupling regime, direct modal switching across a continuous S-shaped dispersion. The resulting figures of merit predict π phase shifts for exciton energy shifts of only a few meV over propagation lengths of tens of micrometers, while larger shifts are still required for mode switching. These results establish multimode waveguide polaritons as a versatile platform spanning multiple coupling regimes, for compact phase and intensity control in integrated photonic architectures.

## Graphical abstract

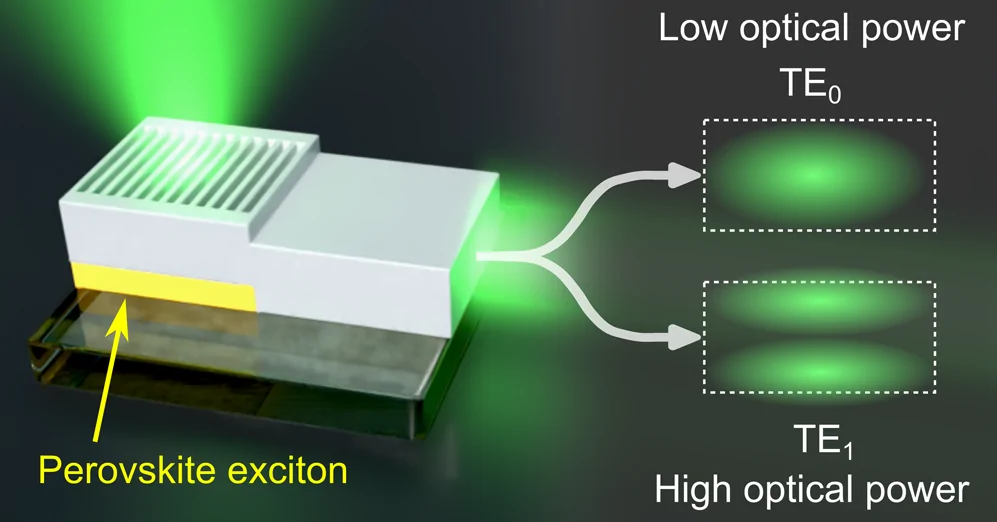

## Introduction

Strong coupling of light and matter has become a ubiquitous phenomenon over the past decades, driven by advances in fabrication, new material discoveries, and innovative photonic designs across a broad range of physical systems. This development led to ever higher coupling strengths between photonic modes from the microwave to visible spectral range and a multitude of coupling regimes and matter excitations, including excitons, phonons, atoms, intersubband transitions, plasmons, and magnons.^1–3^ Such a large light-matter interaction provides experimental access to several coupling regimes with unprecedented physical effects,^4,5^ such as modifications of the vacuum ground state,^6–8^ light-matter decoupling,^9,10^ or altered chemical and material properties.^11–15^ Of particular interest for integrated photonics is the case in which a single material resonance is strongly coupled to multiple photonic modes, producing a multimode polariton dispersion whose branches inherit distinct field profiles and propagation properties from their photonic parents while sharing a common excitonic component. The resulting dispersion provides a rich design space: distinct polariton branches can be co-excited at the same energy, individually addressed by their wavevector, and shifted differentially by perturbations of the matter resonance. Engineering such multimode dispersions across different coupling regimes is the focus of the present work. These regimes range from conventional multimode strong coupling, where each polariton branch retains a dominantly single-mode photonic character, to the so-called superstrong coupling (SSC) regime. In the latter, the Rabi couplings of two or more photonic modes coupled to the same active material become comparable to their mutual energy spacing, leading individual polaritonic branches to acquire contributions from multiple photonic modes.

The SSC region of this design space deserves particular attention because, by enabling coherent hybridization of multiple photonic modes within a single polariton branch,^16,17^ it allows for a dynamical, subwavelength, real-space modulation of the field intensity.^18^ Importantly, SSC can be reached by designing closely spaced photonic modes, without requiring coupling strengths approaching the bare material transition energy, as in the ultrastrong coupling (USC) regime,^19,20^ or the exciton binding energy, as in the very strong coupling regime.^21,22^

SSC was first theoretically described in 2006 for cold atoms trapped in a cavity resonator,^23^ followed by an experimental realization using nearly degenerate transverse modes of a cavity coupled to an atomic ensemble in 2013.^24^ Subsequently, more sophisticated realizations emerged in circuit and cavity QED systems in the microwave domain, coupling longitudinal cavity modes to transmon qubits,^25–27^ acoustic resonators,^28^ or magnon resonances.^29^

All of these realizations have in common the use of long cavities to achieve a small free spectral range, thus reducing the need for very high coupling strengths but requiring the use of high-order modes (mode orders 50–1000) to match the frequency of the matter excitation. However, neighboring high-order modes feature very similar optical properties and field profiles, which makes tuning between such states less interesting from an application perspective. At the other end of the spectrum, realizations in the THz domain use cyclotron resonances of the two-dimensional electron gas in GaAs quantum wells, which provide ultrahigh coupling strengths and thereby enable SSC with low-order modes (mode orders 1–2).^30,31^ However, the requirements for cryogenic temperatures and high magnetic fields limit the broader applicability of these systems.

The visible domain currently presents an interesting gap in the landscape of multimode polariton realizations, as it is the domain of many promising room-temperature excitonic materials for polaritonics, such as perovskites,^32^ TMDs,^33^ and organic dyes.^34^ To date, SSC realizations in this spectral range have focused exclusively on microcavities.^35–38^ While these works achieved SSC with medium-order modes (mode orders 5–12), coupling of the two lowest-order cavity modes has thus far remained elusive due to the unfavorable energy spacing between them: an exciton resonant with the fundamental cavity mode at a given energy would only encounter the second mode at twice that energy because cavity energies depend approximately linearly on the mode order. Reaching SSC with the lowest-order cavity mode would therefore demand coupling strengths in the USC regime, which are difficult to achieve in the visible range.

Here, we address this limitation by replacing microcavities with planar waveguide modes, accessed by a 1D grating, to enable multimode polariton dispersion engineering between the TE_0_ and TE_1_ modes and a room-temperature exciton resonance in the visible spectral range. In contrast to cavities, the mode energies in waveguides are governed by effective refractive indices, which depend on the waveguide thickness and the extent of the evanescent field but only weakly on the mode order. In particular, all effective indices converge toward the bulk refractive index for sufficiently thick waveguides, enabling arbitrarily small energy spacings between the low-order modes. The waveguide thickness thus serves as a single design knob that tunes the system continuously from multimode strong coupling to the SSC regime, hybridizing the distinct field profiles of the two lowest order modes.

For the theoretical description of the waveguide structures, we use a generalized Hopfield model introduced in ref 18 which has been successfully applied to other photonic systems.^30,31^ This work shows how the conditions required to reach SSC can be geometrically engineered. Different electromagnetic modes are in fact orthogonal and therefore induce orthogonal exciton polarization distributions when the full mode volume is occupied by the active material (top row in Figure 1). In this case, the exciton polarization distributions remain independent of each other so that each polariton contains only a single photonic component, making SSC impossible. Creating polaritons with contributions from multiple photonic modes instead requires a high spatial overlap of the photonic mode profiles in the region occupied by the active material. This can be realized by restricting the active material to a subregion of the mode-volume (bottom row of Figure 1), thereby breaking the orthogonality of the modes over the active region and enabling coupling through the non-orthogonal polarization distributions of the excitons.^18^ Early works on SSC naturally achieved these high overlaps since the matter excitation was confined to a negligibly small portion of the long cavities, and therefore this aspect was not discussed explicitly (see Table S1 in the Supporting Information for an overview of representative SSC systems). In contrast, the visible-range implementations we target here require larger fractions of the mode volume to be filled with the active material, making a more careful placement essential, as discussed below. The resulting SSC regime is distinguished, among other features, by a characteristic S-shaped dispersion of the hybrid polariton branch connecting two distinct photonic modes (discussed later in the context of Figures 2 and 3). We note that while high modal overlap is a geometric property accessible at any coupling strength, its physical consequences, coherent hybridization of multiple photonic modes within a single polariton branch, become manifest only when the coupling strength reaches the SSC regime. Below SSC, the same geometry instead produces a multimode strong coupling dispersion with degenerate branches that can be co-excited by a broad-wavevector source, as we exploit in the use cases discussed below.

**Figure 1:**
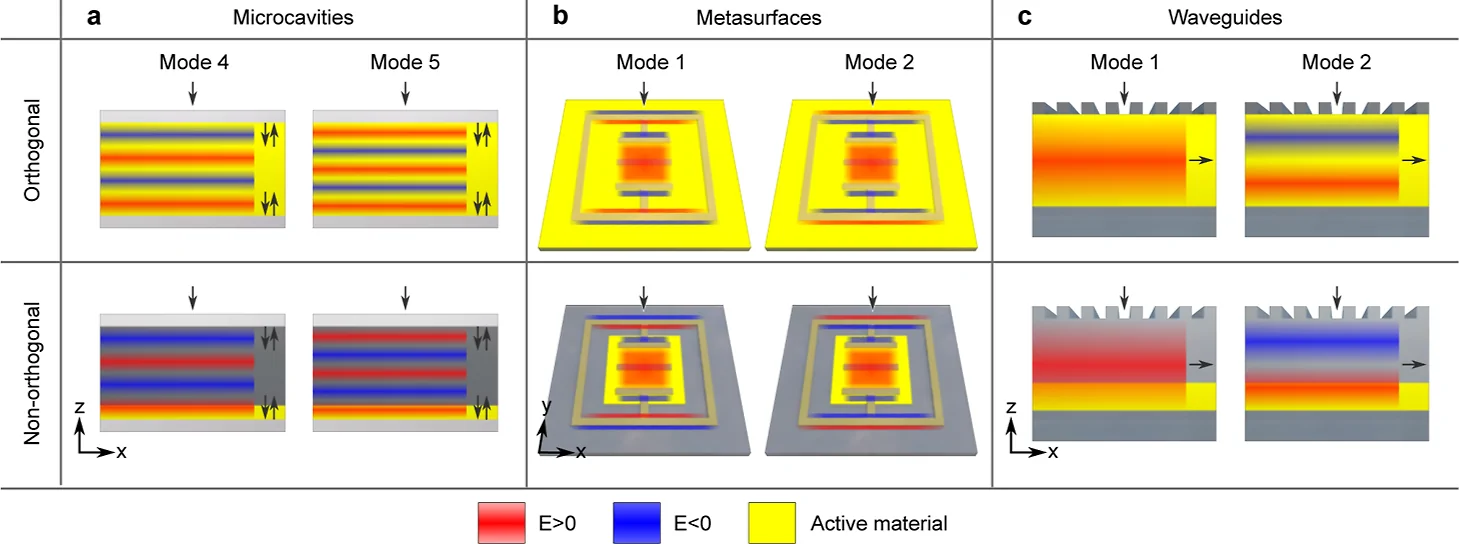
Comparison of platforms for superstrong polariton physics. When the active material (yellow) fills the entire mode volume (upper row), photonic modes are orthogonal over the active region in a Hermitian system, resulting in polariton modes containing only a single photonic mode. Localizing the active material within part of the waveguide (lower row) induces a nonzero overlap between photonic modes over this region, enabling polariton modes with contributions from multiple photonic modes and allowing continuous, coherent tuning between them. (a) Microcavities typically require coupling between higher-order modes to reach the SSC regime, due to the large energy spacing between lower-order modes. (b) Metasurfaces can be engineered for high overlap, but fabrication can be more complex. (c) Waveguides provide both high overlap and low energy separation between lower-order modes, making them ideal for SSC. Blue and red overlays indicate the sign of the electric field, illustrating that positive and negative contributions to the overlap integral cancel in the case of full filling, while partial filling allows for a nonzero overlap.

We note that apart from the waveguides discussed here, SSC with low-order modes can also be realized using metasurfaces,^31^ as summarized in Table 1. However, achieving a high modal overlap in metasurfaces typically requires in-plane patterning of the active material by etching, a process that risks damaging the delicate excitonic materials and adding significant fabrication complexity. Planar waveguides, by contrast, employ the same simple layered deposition used for cavities: the active material is deposited as a thin layer followed by a dielectric spacer without any etching steps on the active layer itself. An overview of these three platforms is provided in Figure 1 and Table 1.

**Table 1:** Comparison of Platforms for Superstrong Polariton Physics

feature	microcavities	metasurfaces	waveguides
modeling	√simple (1D)	X advanced 3D vectorial	√simple (1D/2D)
fabrication	√layered, no etching	X etching, risk of damage	√layered, no etching
mode energy separation	X fixed (1 octave)	√tunable	√tunable
*Q*-factor control	√via mirror reflectivity	√separate for each mode	√via grating thickness
coupling of low-order modes	X generally not possible	√possible	√possible

In this work, we investigate planar waveguides across a range of multimode coupling regimes, using rigorous coupled-wave analysis (RCWA) with realistic parameters of commonly used two-dimensional hybrid organic–inorganic perovskites. By varying the waveguide thickness, we tune the photonic mode spacing and thereby traverse the regime from multimode strong coupling to SSC of the two lowest-order modes. By fitting the generalized Hopfield model from ref 18 to the simulated dispersions, we extract the overlap coefficients and Hopfield coefficients of the polariton modes, confirming the characteristic S-shaped dispersion and the presence of multiple photonic components within individual polariton branches. Building on these results, we demonstrate two complementary use cases:^39^ an interference-based phase modulator that operates in the multimode strong coupling regime, and an exciton-tunable modal switch that exploits the avoided crossing characteristic of the SSC regime.

## Results

### Theoretical Framework

To describe the coupling of multiple photonic modes to a single exciton in our waveguides, we use the recently reported Hopfield-type model introduced in ref 18 briefly summarized here for the case of *N* photonic modes with coupling strengths below the USC regime. A key feature of this model is the introduction of an overlap parameter η_*jk*_ quantifying the normalized spatial overlap between the electric fields **F**_*j*_ and **F**_*k*_ of photonic modes *j* and *k* over the active material region $V_{act}$ as

1 $$\eta_{jk}=\frac{\int_{V_{act}}d^{3}r\;F_{j}^{*}\left(r\right)\cdot F_{k}\left(r\right)}{\sqrt{\left(\int_{V_{act}}d^{3}r\;|F_{j}\left(r\right){|}^{2}\right)\left(\int_{V_{act}}d^{3}r\;|F_{k}\left(r\right){|}^{2}\right)}}$$

An accurate description of arbitrary mode overlap η_*jk*_ requires accounting for the coupling between photonic modes mediated by the exciton. To achieve this, the model introduces *N* degenerate exciton states as auxiliary degrees of freedom, serving as a basis for capturing the effect of the overlap on the coupling between the photonic modes and the single physical exciton state. The resulting Hamiltonian therefore contains 2*N* states (*N* photonic and *N* excitonic), which can be expressed as

2 $$H=\left(\begin{array}{ll} E_{ph} & G\\ G^{†} & E_{ex} \end{array}\right)$$

where the submatrices are given by

3 $$\begin{array}{l} E_{ph}=diag\left(E_{ph,1},...,E_{ph,N}\right),\\ E_{ex}=diag\left(E_{ex},...,E_{ex}\right), \end{array},G=\left(\begin{array}{llll} g_{11} & 0 & \cdots & 0\\ g_{21} & g_{22} & \cdots & 0\\ \vdots & \vdots & \ddots & \vdots \\ g_{N1} & g_{N2} & \cdots & g_{NN} \end{array}\right)$$

where *E*_ph,*j*_ are the energies of the photonic modes, *E*_ex_ is the exciton energy, and *g*_*jk*_ are the coupling strengths between photonic mode *j* and degenerate exciton state *k*. Note that the lower-triangular shape of the coupling strength matrix **G** is a result of the choice of a specific basis for the exciton states, but other forms would be equally valid. The physical exciton fraction of a polariton mode is determined by the sum over all exciton states and is therefore independent of the choice of basis.

The coupling strength matrix *g*_*jk*_ can be related to the previously calculated overlap coefficients via an expansion coefficient matrix α_*jk*_, which determines how the total coupling strength of each photonic mode *j* to the physical exciton *g*_*j*_ is distributed over the degenerate exciton states

4 $$g_{jk}=g_{j}\alpha_{jk},with\;\alpha_{jk}=0\text{for}k>j,\text{and}{\;\eta }_{jk}=\sum_{l=1}^{\min\left(j,k\right)}\alpha_{jl}^{*}\alpha_{kl}$$

This definition automatically ensures normalization of the expansion coefficients as $\sum_{k=1}^{j}|\alpha_{jk}{|}^{2}=1$ , and therefore |α_*jk*_| ∈ [0, 1]. The explicit *N* = 2 form of the model is provided in the Methods section.

Overall, this model generalizes the two commonly used limiting cases, the so-called 2*N* and *N* + 1 models.^35,37,38,40^ When the active material fills the entire mode volume, the overlap matrix reduces to η_*jk*_ = δ_*jk*_, recovering the 2*N* model, where each photonic mode couples to an independent degenerate exciton state. Instead, for an ideal scenario with unit overlap η_*jk*_ = 1, it recovers the *N* + 1 model, where all photonic modes couple to a single exciton, leaving *N* – 1 dark, uncoupled modes.

The model can therefore describe various multimode coupling regimes, with SSC being characterized by at least one pair of photonic modes (*j*, *k*) whose coupling to the same exciton state *l* is comparable to or larger than their energy spacing Δ*E*_ph,*jk*_.

5 $$g_{jl},g_{kl}≃\Delta E_{ph,jk}=\left|E_{ph,k}-E_{ph,j}\right|$$

We stress here that as SSC describes the breaking of the single-mode polaritonic approximation, its violation is a smooth process. As such, the above condition serves as a useful reference for assessing whether the SSC regime can be realized in a specific device, while definitive evidence of SSC is provided by non-negligible contributions from two or more different photonic modes to the same polariton eigenstate, as analyzed in Figures 2 and 3.

One way to fulfil the SSC condition, is through a high spatial overlap (η_*jk*_ ≈ 1) in combination with high total coupling strengths *g*_*j*_ and *g*_*k*_ ≃Δ*E*_ph,*jk*_. Such a high overlap directly shapes the polariton dispersion. In the perfect overlap limit of η_*jk*_ = 1, the energy gap between pairs of upper and lower polariton branches originating from different photonic modes closes, yielding a characteristic S-shaped dispersion in which it is possible to continuously tune the polariton from one dominant photonic character to the other (see an example of such a dispersion in Figure 2). In the realistic cases considered here, where η_*jk*_ < 1, a finite gap Δ*E*_pol,*jk*_ opens at the crossover between polaritons *j* and *k*, but remains insignificant as long as it is small compared with the linewidths of the involved polaritons, in our case dominated by the exciton linewidth γ_ex_. As shown for the *N* = 2 case in Section S5 of the Supporting Information, a sufficiently small energy gap can be realized both by large overlaps or by low coupling strengths. Therefore, it is not possible to define a general overlap threshold for achieving continuous tuning in the SSC regime.

In the following, we fit the model to the polariton dispersion from RCWA simulations with *E*_ex_ and *E*_ph,*j*_ fixed, using a total of *N*(*N* + 1)/2 fitting parameters: *N* real coupling strengths *g*_*j*_ and *N*(*N* – 1)/2 real-valued α_*jk*_ parameters with the normalization ensured by a hyperspherical parameterization. Note that only bound waveguide modes are included, as the quantities extracted from the model depend only on the fields within the waveguide core, where the guided-mode basis provides an accurate description. A complete reconstruction of the electromagnetic field outside the core would additionally require including the radiation continuum. The eigenvectors yield the Hopfield coefficients (fractions) for the photonic modes |*C*_*j*_|^2^ and the total exciton fraction ${\left|X\right|}^{2}=\sum_{j=1}^{N}{\left|X_{j}\right|}^{2}$ of each polariton, allowing us to confirm the presence of multiple photonic modes inside a polariton mode as well as the possibility to continuously tune between them.

We now apply this general theoretical framework to the case of planar waveguide geometry.

### Ideal Waveguide Model

To gain an understanding of how to achieve high modal overlap, we first analyze an idealized system amenable to an analytical treatment: two waveguide modes in a planar waveguide of total thickness *D* with perfectly reflecting walls (see Section S6 of the Supporting Information for details), resulting in the following field profiles

6 $$F_{j}\left(x,z\right)=\sin\left(\frac{j\pi z}{D}\right)ŷ$$

Note that we use *j* = 1 for the TE_0_ mode and *j* = 2 for the TE_1_ mode throughout, i.e., the index *j* corresponds to the TE_*j*–1_ waveguide mode.

We optimize both the position and the volume fraction Δ*z*/*D* of the active material layer of thickness Δ*z* (see geometry in Figure 2a). The optimization is performed by directly calculating the overlap integral of the electric fields **F** (eq 1) to maximize the geometric mean of the coupling strengths, $\sqrt{g_{1}g_{2}}$ , for a given target overlap η_12_.

**Figure 2:**
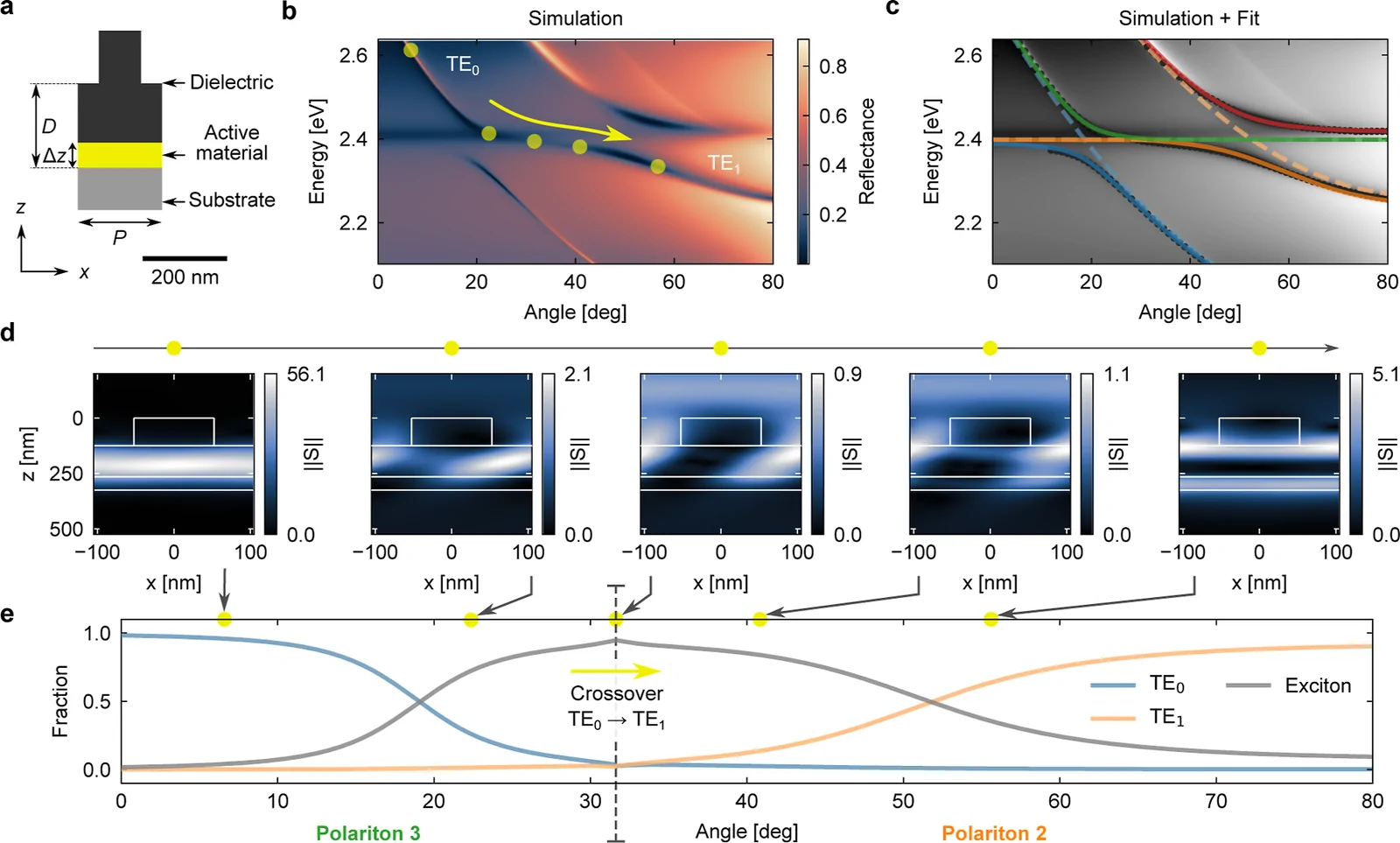
Simulation of multimode strong coupling with high modal overlap in the low-thickness waveguide (*D* = 200 nm). (a) Unit cell of the grating structure with active material fraction Δ*z*/*D* = 0.3 and periodicity *P* = 210 nm. (b) Polariton dispersion in reflection. (c) Fit using TE_0_ (blue dashed) and TE_1_ (orange dashed) photonic modes, yielding four polariton branches. Small gray dots below the colored curves indicate points used in fit. (d) Poynting vector magnitude at selected yellow points in (b), illustrating the crossover from TE_0_ to TE_1_. (e) Fractions of uncoupled modes. Polariton 2 (orange) is shown to the right and polariton 3 (green) to the left of the crossover line (gray dashed), which marks the angle where the energy gap between the polaritons is minimal. Due to the small energy gap, no significant discontinuity is observed in the fractions. All evaluated quantities of this waveguide are listed in Table 2.

The results of this optimization reveal a simple design principle: for maximizing the coupling strength at any desired overlap, the optimal placement for a single layer of active material is either at the bottom or at the top of the waveguide. The thickness of this layer then determines the overlap with thinner layers yielding higher overlaps. Intuitively, for an infinitesimally thin layer at the waveguide boundary, the overlap approaches unity, because ideal waveguide modes vanish at the boundaries and can be approximated as linear functions with different slopes. In this limit, the modes are identical up to a scaling factor, resulting in unity overlap, which we remind is a normalized dimensionless quantity.

However, as the field strength is low near the outermost positions, the coupling strength is also low, thus requiring a trade-off between overlap and coupling strength. An active material fraction of Δ*z*/*D* = 0.3 was chosen for the numerical simulations, providing an overlap of η_12_ = 0.99 while still maintaining a high coupling strength.

### Waveguide Design and Simulation

Based on these theoretical considerations, we design a grating-coupled planar waveguide structure to achieve SSC in the visible range. The key advantage of waveguides is that the photonic mode energy spacing can be tuned without significantly affecting the energy of the fundamental TE_0_ mode. Specifically, in the limit where the waveguide is much thicker than the wavelength (see Methods section), the mode energies can be approximated as

7 $$E_{j}\left(\beta \right)\approx \frac{\hbar c}{n_{0}}\beta \left[1+\underset{\ll 1}{\underset{︸}{\frac{\pi^{2}n_{0}^{2}}{2\beta^{2}}\frac{j^{2}}{D^{2}}}}\right]$$

where *n*_0_ is the refractive index of the waveguide material and β is the in-plane wavevector (propagation constant) of the waveguide mode. Note that the mode energy depends only weakly on the mode order *j*, while the energy spacing between modes scales as 1/*D* ^2^ and can therefore be tuned as needed, down to the point of near-degeneracy. In contrast to microcavities where the mode spacing is fixed to the fundamental mode energy, this enables exploring SSC with the two lowest-order modes (TE_0_ and TE_1_), which offers two advantages: (1) the modes have more distinct field profiles and thus more distinct optical properties; and (2) as shown later in Figure 4, for a given desired overlap, the achievable coupling strength is significantly higher than for higher-order mode pairs.

In order to demonstrate the tunability of the mode energy spacing, we focus on three different waveguide geometries with different core thicknesses in the rest of the paper: low- (*D* = 200 nm), mid- (*D* = 400 nm), and high-thickness (*D* = 600 nm) waveguides. Each thickness serves a distinct role in the analysis that follows: the 200 nm waveguide illustrates the multimode strong coupling regime below SSC and underpins the interference-based phase modulator; the 400 nm waveguide reaches the SSC regime proper and underpins the modal-switching scheme; and the 600 nm waveguide supports five photonic modes simultaneously and is used for the systematic parameter sweep over active-material fraction and mode order in Figure 4. As shown in Figure 2a, we place the active material of thickness Δ*z* as bottom-most layer of the waveguide core, comprising a fraction Δ*z*/*D* = 0.3 of the total core thickness, as motivated by the optimization results for ideal waveguides. As parameters for our active material, we choose values similar to the A-exciton in common two-dimensional hybrid organic–inorganic perovskite crystals^41^ with a resonance in the visible at 517 nm (*E*_ex_ = 2.398 eV). We note that with these parameters, the TE_0_ and TE_1_ modes of all studied waveguides fulfill the strong coupling condition, as verified in Table 2. For coupling light into the waveguide modes, we consider a one-dimensional grating on top of the waveguide core. More details on the materials and geometric parameters are available in the Methods Section.

We then use rigorous coupled-wave analysis (RCWA, S4 ^42^) to simulate the reflectance of the structure as a function of the incidence angle and extract the polariton dispersion. Details of the simulation and extraction procedure are provided in Section S3 of the Supporting Information. The resulting dispersion is fitted using the model described in the previous section to retrieve the overlap coefficients and Hopfield coefficients of the polariton modes.

In this context, we also point out that the number of photonic modes required for an accurate theoretical description is naturally limited for two reasons. First, the overlap integral between the fundamental mode and a higher-order mode *j* remains large only for modes whose field varies slowly across the active layer, i.e., for *j* ≲ *D*/Δ*z*. Second, according to eq 7, the energy spacing between adjacent waveguide modes increases approximately linearly with *j*, in contrast to the constant energy spacing of microcavities. As a result, the contribution of progressively higher-order modes is suppressed both by their decreasing overlap with the active layer and by their increasing energetic detuning.

### Superstrong Coupling in Simulated Structures

We start by analyzing the thin waveguide (*D* = 200 nm), deliberately chosen to yield a large mode energy spacing that places the system in the multimode strong coupling regime below SSC, with *g*_11_/Δ*E*_ph,12_ = 0.11 and *g*_21_/Δ*E*_ph,12_ = 0.12. The simulation results in Figure 2 exhibit the characteristic S-shaped dispersion, enabling continuous tuning between polariton modes dominated by TE_0_ and TE_1_ as the incidence angle is varied.

Fitting the Hopfield model (*N* = 2) produces four polariton branches that closely match the simulated dispersion, with a root-mean-square error of 3.6 meV per point, well below the linewidths of the relevant modes (see Table 2). The high overlap η_12_ = 0.99 results in a negligibly small energy gap of 3 meV between the two inner polariton branches (labeled 2 (orange) and 3 (green) in Figure 2), well below the polariton linewidths. This high overlap, combined with the moderate coupling strength, places the system in a multimode strong coupling regime: the dispersion exhibits avoided crossings, but the individual polariton branches remain dominantly composed of a single photonic mode, wherever the photonic fraction is nonnegligible, as confirmed by the Hopfield coefficients in Figure 2e.

As a consequence of their non-SSC nature, the polariton modes are dominantly exciton-like at the crossover point, leading to a weak electromagnetic field confinement (see maximal Poynting vector values in Figure 2d). This device will be exploited for the interference-based phase modulation, where the two branches are co-excited by a broad-wavevector source. Increasing the waveguide thickness while keeping the active-material fraction fixed at 30% reduces the mode energy spacing without significantly affecting the overlap. For the mid-thickness waveguide (*D* = 400 nm, Figure 3), this increase in thickness therefore drives the system into the SSC regime with *g*_11_/Δ*E*_ph,12_ = 0.26 and *g*_21_/Δ*E*_ph,12_ = 0.37, while maintaining the high overlap of η_12_ = 0.97. Due to the higher coupling strength, the energy gap between the two inner polariton branches at the TE_0_–TE_1_ crossover point increases to 13.8 meV, which is still below the polariton linewidths, dominated by the exciton linewidth γ_ex_ = 30 meV, and thus sufficiently small for allowing a continuous transition.

**Figure 3:**
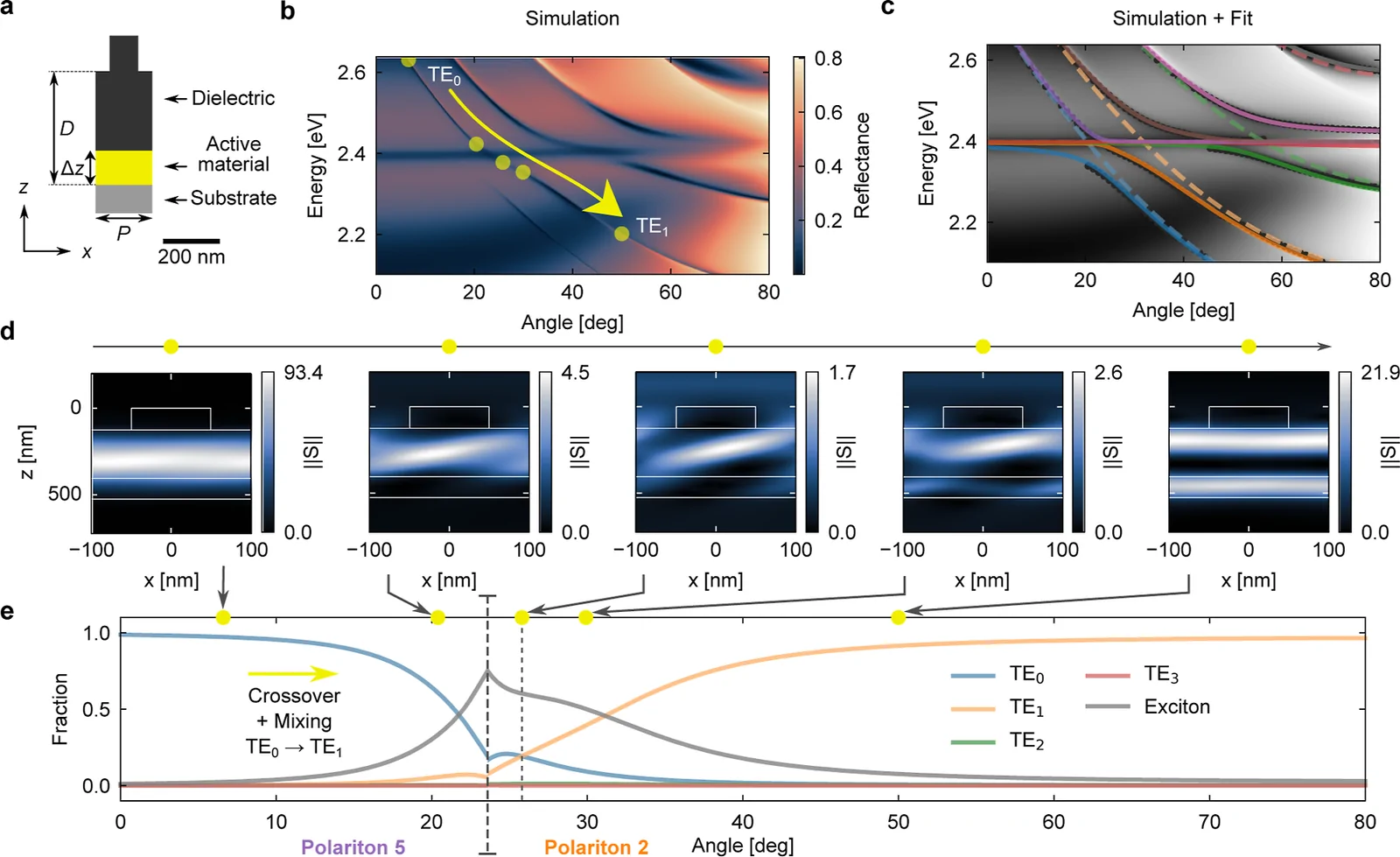
Simulation of superstrong coupling in the mid-thickness waveguide (*D* = 400 nm). (a) Unit cell of the grating structure with active material fraction Δ*z*/*D* = 0.3 and periodicity *P* = 200 nm. (b) Polariton dispersion in reflection. (c) Fit using TE_0_–TE_3_ photonic modes (dashed lines), yielding 8 polariton branches. Small gray dots below the colored curves indicate points used in fit. (d) Poynting vector magnitude at selected yellow points in (b), illustrating the crossover from TE_0_ to TE_1_. (e) Fractions of uncoupled modes. Polariton 2 (orange) is shown to the right and polariton 5 (purple) to the left of the crossover line (gray dashed), which marks the angle where the energy gap between the polaritons is minimal. Due to the small energy gap, no significant discontinuity is observed in the fractions. All evaluated quantities of this waveguide are listed in Table 2.

Analysis of the Hopfield coefficients of the polariton modes confirms the presence of both photonic modes in each polariton branch as well as the continuous tuning between them across the crossover point (see Figure 3e). Furthermore, the increased coupling strength results in a significantly higher photonic fraction at the crossover point, leading to a better field confinement and a higher reflection contrast. Contrary to the thin waveguide, this thicker geometry therefore allows the creation of coherent superposition states of the two photonic modes in reflection, even with an incoherent excitation source with the coherence provided by the mutual coupling to the exciton.

We note that in thicker waveguides, the TE_0_-dominated polariton mode profile slightly retreats from the active material layer at energies above the exciton resonance (see Figure 3d, second field profile). This effect arises because the real part of the refractive index of the active material becomes lower than that of the dielectric filler near the exciton resonance, causing the active layer to act as a low-index region and thus repel the mode. Importantly, this shift does not significantly affect the coupling strength, which remains comparable to that of the thin waveguide (cf. Table 2).

Lastly, we investigate how the fraction of active material influences the crossover behavior and compare the performance of coupling the lowest-order modes (TE_0_/TE_1_) with that of higher-order modes (TE_3_/TE_4_). For this analysis, we use the thick (*D* = 600 nm) waveguide, which enables simultaneous observation of the dispersion for TE_0_–TE_4_ modes. The results of the fits for various active material fractions Δ*z*/*D* ranging from 0.05 to 1 are presented in Figure 4, with the complete characterization equivalent of Figures 2 and 3 provided in Section S4.3 of the Supporting Information.

**Figure 4:**
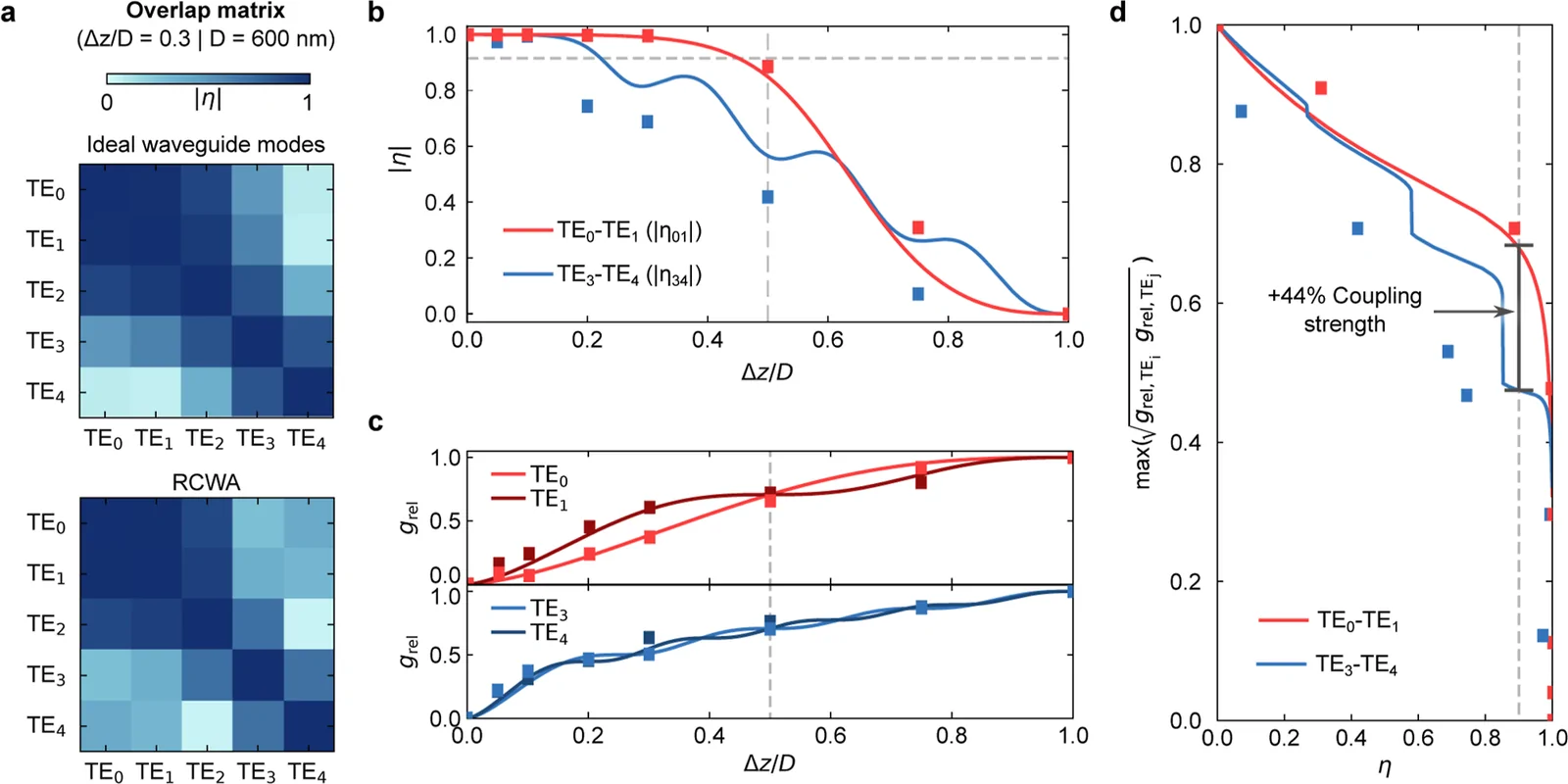
Impact of active material fraction on crossover behavior for the high-thickness waveguide (600 nm). (a) Example of an overlap matrix η_*jk*_ at an active material fraction Δ*z*/*D* = 0.3. All evaluated quantities of this waveguide are listed in Table 2. (b) Overlap between TE_0_/TE_1_ and TE_3_/TE_4_ decreases with increasing active material fraction Δ*z*/*D*. (c) Conversely, the total relative coupling strengths *g*_rel,*j*_(Δ*z*) = *g*_*j*_(Δ*z*)/*g*_*j*_(*D*) of photonic modes to excitons increase. (d) Maximally achievable geometric mean of relative coupling strengths for a given desired overlap for ideal waveguide modes (eq 6), highlighting an increased coupling strength for TE_0_/TE_1_ modes compared to higher-order modes. All values shown in this figure are obtained using the Hamiltonian model, with lines indicating results using eq 6, and rectangles indicating fits to simulated RCWA data. Full fit results and additional details provided in Section S6 of the Supporting Information.

The theoretical model of ideal waveguide modes provides a reasonable estimate of the overlap matrix η_*jk*_, with the fitted overlap values following the direct calculation of the overlap integral using eq 6 (see Figure 4a). This agreement confirms both the validity of the theoretical model and the successful extraction of the overlaps from the fit. For the active material fraction Δ*z*/*D* = 0.3, we find η_12_ = 0.995 (ideal: 0.990), η_14_ = 0.879 (ideal: 0.893), and η_45_ = 0.687 (ideal: 0.816), with values for other active material fractions shown in Figure 4b. The ideal waveguide model (eq 6) also predicts the coupling strength, normalized to the value for a completely filled waveguide, *g*_rel_, as a function of the active material fraction (see Figure 4c), using that $g_{j}\propto \sqrt{\int dz|F_{j}\left(z\right){|}^{2}}$ . As shown in Figure 4c, these predictions closely match the values extracted from the RCWA fits. In this context, we also note that restricting the Hamiltonian to the first five waveguide modes is justified, even for the high ratio of coupling strength to mode spacing *g*_2_/Δ*E*_ph,12_ = 0.74 in the thick waveguide. As demonstrated in Figures S4 and S5 of the Supporting Information, each of the ten polariton branches contains, to good approximation, contributions from no more than two photonic modes at any given detuning. Therefore, modes of even higher order can be safely neglected.

Two important results emerge from this analysis. (1) There is a fundamental trade-off between coupling strength and overlap: increasing the active material fraction increases the coupling strength but reduces the overlap. (2) For a given desired overlap, the achievable coupling strength can be significantly higher when using the lower-order modes (TE_0_/TE_1_) compared to higher-order modes (TE_3_/TE_4_), as shown in Figure 4d. In particular, to achieve a high overlap of η_*jk*_ = 0.9, the maximally achievable coupling strength for TE_0_/TE_1_ modes is 44% higher than for TE_3_/TE_4_ modes under ideal placement of the active material. This is because the rapid spatial oscillations of higher-order modes lead to the cancellation of positive and negative contributions to the overlap integral, thus requiring a thinner layer to achieve the same overlap, which in turn reduces the coupling strength. Note that these two results apply equally to cavities, as their idealized spatial mode profiles are identical to those of waveguides. However, coupling between TE_0_ and TE_1_ modes is generally not feasible in cavities due to their large mode energy spacing, which highlights a key advantage of waveguides in realizing superstrong coupling.

**Table 2:** Comparison of Simulated Waveguide Structures for an Active-Material Fraction Δ*z*/*D* = 0.3^a^

*D*	*P*	*g*_*j*_	Δ*E*_ph,12_	*g*_*j*_/Δ*E*_ph,12_	η_12_	Δ*E*_pol,*jk*_	γ_ex_	γ_TE_0__	γ_TE_1__	SC_*j*_	fit RMS
[nm]	[nm]	[meV]	[meV]			[meV]	[meV]	[meV]	[meV]		[meV]
200	210	44–49	391	0.11–0.13	0.990	3.0	30	8.5	29.7	2.00–1.64	3.6
400	200	40–58	152	0.26–0.38	0.972	13.8	30	1.6	4.2	1.88–2.71	4.2
600	200	37–59	80	0.46–0.74	0.995	2.6–10.1	30	0.9	1.9	1.74–2.78	2.7

a Shown are the waveguide thickness *D*, grating period *P*, TE_0_–TE_1_ energy spacing Δ*E*_ph,12_, coupling strength *g*_*j*_ (first value for TE_0_, second for TE_1_), modal overlap η_12_, fit root-mean-square error per point, full width at half-maximum linewidths γ_ex_ (exciton) and γ_ph,*j*_ (photonic modes), the polariton energy gap at crossover Δ*E*_pol,*jk*_ (two values indicate two gaps exist), and the strong coupling criterion for the two photonic modes ${SC}_{j}=g_{j}/\sqrt{\left(\gamma_{ex}^{2}+\gamma_{{TE}_{j-1}}^{2}\right)/2}$ ^63^ (first value for TE_0_ and second for TE_1_).

## Discussion

In this section, we explore different use cases for the multimode waveguide platform we have described and characterized. The first relies on interference between two polariton modes in the multimode strong-coupling regime to achieve phase modulation by shifting the exciton resonance. The second exploits switching between two photonic modes by sweeping the exciton energy across the avoided crossing.

### Interference-Based Phase Modulation in the Multimode Strong Coupling Regime

We first demonstrate that even modest exciton energy shifts of less than 10 meV enable mode switching via interference between two polariton modes. The key observation is that two photonic modes strongly coupled to a single exciton result in a dispersion that allows two polariton modes to be excited at the same energy but with distinct wavevectors that respond differently to changes in *E*_ex_. As the polaritons propagate, they accumulate a phase difference ΔΦ that can be tuned by adjusting *E*_ex_, thereby modulating their interference at the output.

In Figure 5, we illustrate this mechanism for the low-thickness waveguide. Here, a focused laser with a broad wavevector distribution would coherently excite both polariton 1 and polariton 2 at a fixed energy *E*_Laser_ (white horizontal line). We then evaluate how the wavevectors β_1_ and β_2_ of these two polariton modes shift as a function of the exciton energy *E*_ex_. To quantify the sensitivity of each polariton for small shifts in the exciton energy, we use the value of the slope ∂β/∂*E*_ex_. The difference between these slopes then defines the figure of merit (FOM) for interference-based switching

8 $$FOM=\left|\frac{\partial \beta_{1}}{\partial E_{ex}}-\frac{\partial \beta_{2}}{\partial E_{ex}}\right|,,\Delta \Phi_{ex}=FOM\Delta E_{ex}L$$

where Δ*E*_ex_ is the exciton energy shift, *L* is the fixed propagation distance in the waveguide, and ΔΦ_ex_ denotes the tunable phase shift caused by Δ*E*_ex_, which adds to the static term ΔΦ_0_ = (β_1_ – β_2_)*L* evaluated at Δ*E*_ex_ = 0.

**Figure 5:**
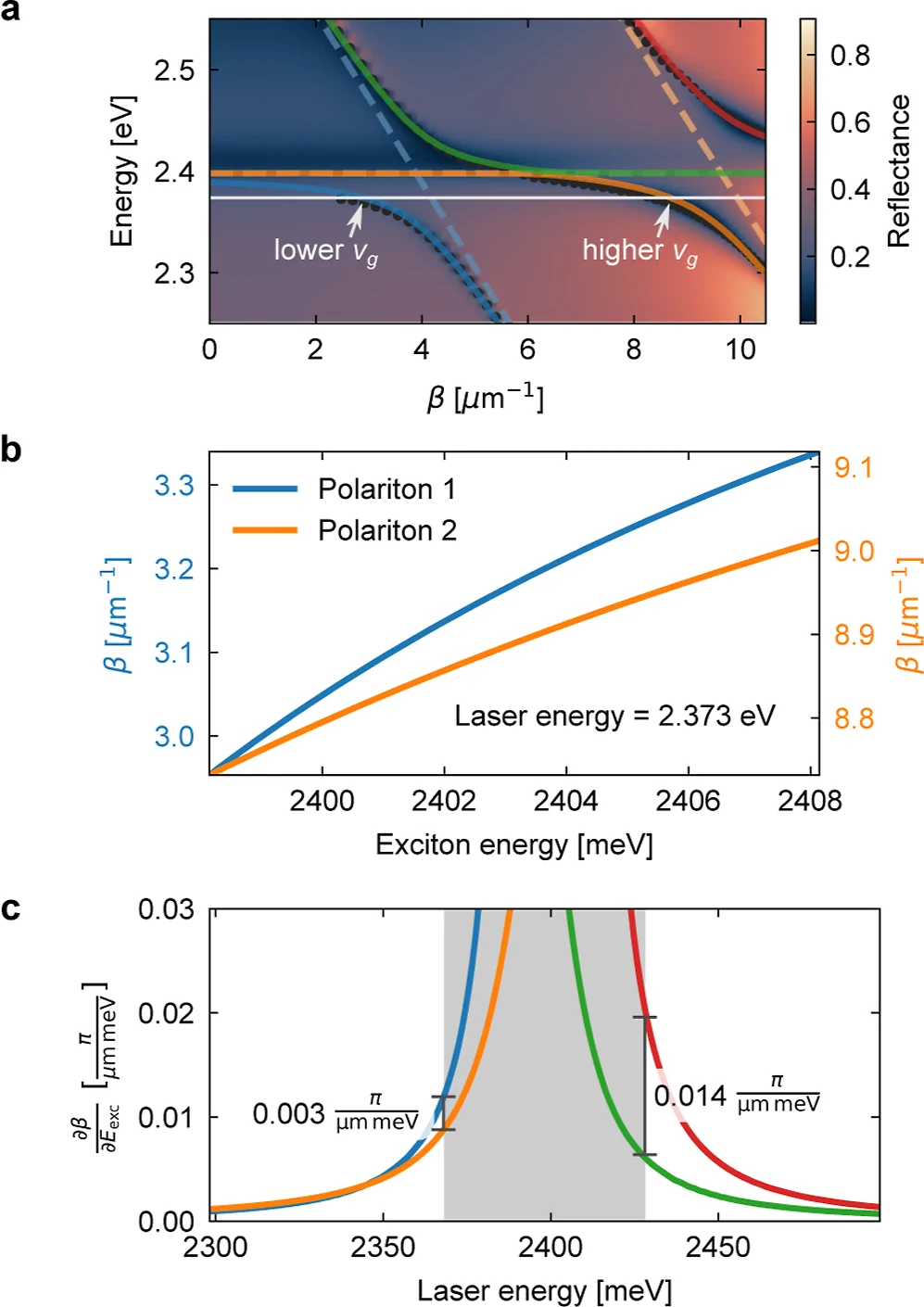
Exciton-tunable interference-based switching in the low-thickness waveguide (*D* = 200 nm, Δ*z*/*D* = 0.3). (a) Dispersion of the waveguide from Figure 2 as a function of in-plane wavevector β. A focused laser at fixed energy (white horizontal line) coherently excites both polariton 1 (blue, TE_0_) and polariton 2 (orange, TE_1_) due to its broad wavevector distribution. (b) Tuning the exciton energy *E*_ex_ leads to distinct shifts in β for the two polaritons due to their different exciton fractions and group velocities. (c) Magnitude of the wavevector shift, shown as *∂*β/*∂E*_ex_ evaluated at the original exciton energy (2.398 eV). The difference between these derivatives for the two polariton modes quantifies the phase shift per unit energy shift and propagation length, serving as the FOM for this switching scheme. The FOM increases as the laser energy approaches the exciton resonance. The gray shaded area indicates an energy range of 2γ_ex_ centered at the exciton, where the system is too lossy for operation. All evaluated quantities of this waveguide are listed in Table 2.

To gain physical insight into this mechanism, we note that in the low-thickness waveguide, where the two polariton modes are predominantly composed of a single photonic mode, a 2 × 2 Hopfield model yields the closed-form result (derived in Section S7 of the Supporting Information)

9 $$\frac{\partial \beta_{j}}{\partial E_{ex}}=-\frac{|X_{j}{|}^{2}}{\hbar v_{g,j}}$$

where |*X*_*j*_|^2^ is the exciton fraction and *v*_*g*,*j*_ is the group velocity of polariton mode *j*, as shown in Figure S17 of the Supporting Information. While the results from this 2 × 2 Hamiltonian are instructive for guiding the design, we point out that we use the full multi-mode Hamiltonian (eq 2) and directly calculate the derivatives with eq 8 in the following.

As shown in Figure 5c, the FOM increases as the laser energy approaches the exciton resonance. This enhancement is driven by both the higher excitonic fraction and the reduced group velocity of the polariton modes near resonance, as described by eq 9. In order to avoid the excessive losses from the exciton, we determine the FOM at a laser detuning of *E*_Laser_ – *E*_ex_ = γ_ex_ from the exciton. The FOM reaches 3 × 10^–3^ π meV^–1^ μm^–1^ for the lower polariton branches, so that a blueshift of 5 meV is sufficient for a π phase shift after 67 μm of propagation. For the upper polariton branches, the FOM can reach 14 × 10^–3^ π meV^–1^ μm^–1^, reducing the required propagation length to 14 μm for the same blueshift.

Active-material position and thickness, as well as the overall waveguide thickness, provide additional free parameters for engineering a dispersion with even larger differences in wavevector sensitivity between the two polariton modes, thus further optimizing the FOM. Nevertheless, the energy shifts and propagation distances required by the present proposal are already within experimental capabilities.^43^ Comparable switching lengths down to 20 μm have been reported in slow-light Mach–Zehnder interferometers and bimodal waveguides in silicon photonics,^44,45^ but electro-optical and thermo-optical modulation in those platforms limits the achievable switching speed. All-optical switching can overcome this limitation with π phase shifts having been demonstrated in a comparably sized polaritonic Mach–Zehnder geometry with potential switching speeds of ∼100 ps in a GaAs platform.^46^ However, that approach requires spatially localized optical pumping of one of the arms of the interferometer. The multimode waveguide geometry proposed here relaxes this constraint, since both polariton modes are co-excited by a single spatially uniform pump, eliminating the need for strict alignment of the excitation.

### Continuous Real-Space Field Reshaping and Modal Switching in the SSC Regime

Having demonstrated phase control in the multimode strong coupling regime, we now turn to the SSC regime, where the avoided-crossing topology enables qualitatively different modes of operation. Near the crossover point of the mid-thickness waveguide (*D* = 400 nm, Δ*z*/*D* = 0.3), well into the SSC regime, polariton modes contain significant contributions from both TE_0_ and TE_1_ modes. This coherent superposition state would form even under incoherent illumination, as the coherence is inherited from the mutual coupling to the exciton. The resulting electromagnetic state can be written as

10 $$|\psi \rangle =\cos\theta |{TE}_{0}\rangle +e^{i\phi }\sin\theta |{TE}_{1}\rangle$$

By adjusting the exciton energy, one obtains continuous control over the amplitude ratio of the two modal components, setting the angles θ and ϕ. Crucially, because |TE_0_⟩ and |TE_1_⟩ have distinct transverse field profiles, this continuous tuning of θ translates directly into a continuous reshaping of the electromagnetic mode profile in real space, enabling sub-wavelength control of the field concentration across the active layer. The device therefore acts as a waveguide-integrated beamsplitter in the modal degree of freedom and, equivalently, as a continuously tunable real-space field-shaping element, opening the possibility of switches based not only on frequency or output channel but also on the spatial concentration of the field itself. In the single-photon regime, the structure would prepare arbitrary qubit states encoded in the transverse mode of the waveguide, providing a compact alternative to path encoding^47,48^ that requires only a single physical waveguide rather than two spatially separated paths. Combined with mode-dependent output couplers, this enables tunable on-chip state preparation and measurement within a simple planar geometry. The modal-switching limit of this continuous reshaping is illustrated in Figure 6, where the SSC regime enables a continuous crossover between TE_0_-like and TE_1_-like polaritons as a function of the exciton energy at a fixed incidence angle and fixed excitation energy interval.

**Figure 6:**
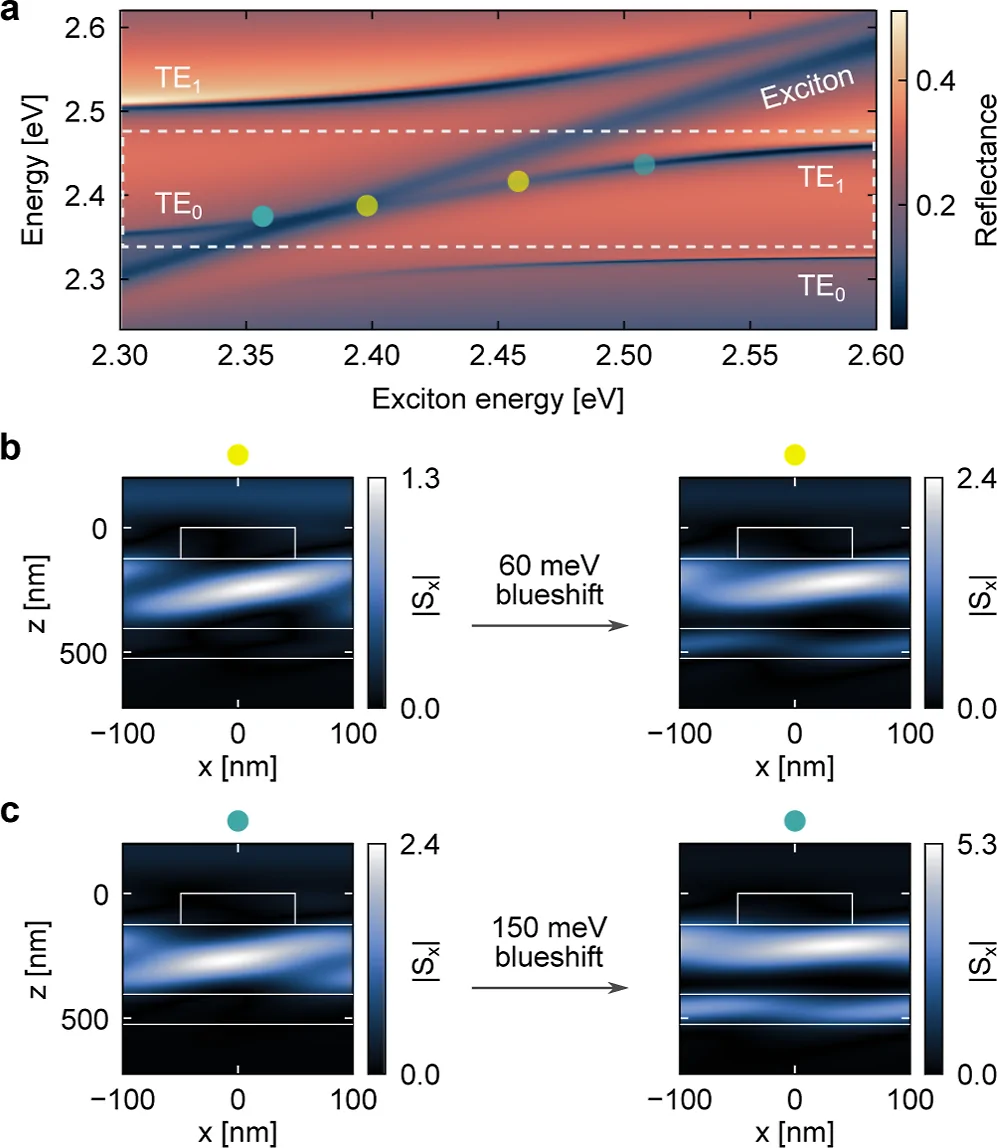
Exciton-tunable mode switching for the mid-thickness waveguide (*D* = 400 nm, Δ*z*/*D* = 0.3). For this application, the excitation is fixed at an incidence angle of 22° and would cover the energy range of the two inner polaritons (gray dashed rectangle), ensuring that for any given exciton energy only one polariton mode is excited. (a) Polariton energy in reflection as the exciton energy is varied. (b,c) Magnitude of the *x* component of the Poynting vector at the selected yellow (b) and green (c) points shown in (a), illustrating the crossover from TE_0_ to TE_1_ achievable with different amounts of exciton blueshift. All evaluated quantities of this waveguide are listed in Table 2.

While the required energy shifts of 60–150 meV are comparatively high in our example, further structural optimization could reduce the requirements, such that commonly achievable exciton energy shifts of tens of meV would suffice, e.g., through nonlinear effects (phase-space filling, exciton–exciton interactions, bandgap renormalization, and optical Stark effect),^49^ temperature variation,^50^ or electrostatic gating of the excitonic material.^51^ Such improvements can be achieved by further increasing the waveguide thickness, which reduces the mode energy spacing and, thereby, lowers the required exciton energy shift. However, we chose the mid-thickness waveguide for this illustration because it offers the best reflection contrast at the given grating thickness, whereas thicker waveguides require a redesigned grating with stronger far-field coupling to maintain sufficient contrast. Overall, the presented setup effectively realizes a waveguide-integrated mode switch, routing light between two distinct output channels and offering a polaritonic alternative to nonlinear directional couplers.

## Conclusion

In this work, we introduced planar waveguides as a platform for multimode polariton dispersion engineering, spanning the regime from multimode strong coupling to superstrong coupling between an exciton and multiple photonic modes in the visible spectral range. The SSC end point of this range is characterized by polaritons with multiple photonic components and a characteristic S-shaped dispersion. The weaker multimode strong coupling regime, accessed at a smaller waveguide thickness, retains a single-photonic-mode character per polariton branch but provides the differential branch sensitivity exploited in our phase-modulation scheme.

By applying a generalized Hopfield model^18^ to both ideal waveguide modes and RCWA simulation results, we showed that the optimal strategy for maximizing the coupling strength at a given overlap is to place the active material at the upper or lower boundary of the waveguide core. An active material fraction of Δ*z*/*D* = 0.3 was found to provide a good compromise between substantial coupling strength and a high overlap of η_*jk*_ ≈ 0.99.

We further demonstrated that waveguides offer a key advantage over microcavities in that the energy spacing between photonic modes can be freely tuned by adjusting the waveguide thickness without significantly affecting the fundamental mode energy. We investigated three waveguide thicknesses (*D* = 200, 400, and 600 nm), spanning the range from multimode strong coupling to SSC (*g*_*j*_/Δ*E*_ph,12_ up to 0.74). This tunability enables coupling of the two lowest-order modes (TE_0_ and TE_1_), which is not generally feasible in cavities due to their large mode spacing. Coupling of these lower-order modes leads to up to 44% higher coupling strengths for a given overlap compared to higher-order mode pairs (TE_3_/TE_4_), while also providing more distinct field profiles.

All simulations were performed using realistic parameters for two-dimensional hybrid organic–inorganic perovskites (e.g., F-PEA), which are compatible with the proposed layered structure and represent a promising candidate for experimental realization. The waveguide approach can be extended to other excitonic materials (TMDs^52^ and organic dyes^53^) to coupling between modes of different polarization or propagation loss or to hybrid plasmonic-photonic systems.^54^

While further optimization of material parameters, waveguide geometry, and fabrication strategy is needed, the requirements are well within reach of current experimental capabilities and are based on established fabrication techniques.^43,55–57^ Compared to cavities and metasurfaces, the in-plane propagation of waveguide modes offers the advantage of being directly compatible with existing on-chip photonic components. This positions planar waveguides as a promising route toward integrated photonic applications, such as ultrafast mode switching driven by transient exciton energy shifts and the generation of coherent modal superposition states for quantum photonic circuits. Overall, these results establish planar waveguides as a versatile, scalable platform for translating superstrong coupling physics from fundamental research into practical integrated photonic devices at visible wavelengths and room temperature.

## Methods

### Waveguide Materials and Design

As parameters for the active material layer in the waveguide, we choose values comparable to common two-dimensional hybrid organic–inorganic perovskite crystals,^41^ with a constant background refractive index of 2.3 (ϵ_∞_ = 2.3^2^), a single Lorentzian oscillator with a resonance at 517 nm (*E*_ex_ = 2.398 eV), an oscillator strength corresponding to *ℏ*ω_p_ = 0.5 eV, and a FWHM of γ_ex_ = 30 meV (see definition for Lorentzian material in Section S2 of the Supporting Information). Recent experiments have shown that it is possible to grow uniform single crystals of 4-fluoro-phenethylammonium lead iodide (F-PEA) perovskite with the required thicknesses and optical properties,^32,58,59^ making it a promising candidate for experimental realization of the proposed structures. Another suitable material candidate among 2D perovskites is phenylethylammonium lead iodide (PEPI), for which strong coupling has been repeatedly demonstrated.^60–62^

The remainder of the core is filled with a lossless dielectric that matches the background refractive index of the active material (*n*_0_ = 2.3). This can be achieved experimentally by depositing a TiO_2_ layer via electron beam evaporation. On top of the waveguide, we define a 125 nm thick grating layer composed of the same lossless dielectric with a filling factor of 0.5. This grating enables the efficient coupling of light into the waveguide modes from above. Experimentally, the grating layer can be fabricated by electron-beam lithography, which minimizes damage to the perovskite crystal as it is protected by the TiO_2_ layer during this step.

The thickness of the grating was adjusted to tune the *Q*-factor of the photonic modes. For optimal coupling, it must be thick enough to ensure a strong interaction (i.e., lowering the photonic *Q*-factor toward that of the exciton), yet thin enough to avoid a significant alteration of the mode profiles. The grating periodicity is set to *P* = 200–210 nm, enabling coupling to the TE_0_ and TE_1_ modes at experimentally accessible angles of incidence. The waveguide is cladded below by a semi-infinite SiO_2_ substrate with a constant refractive index of 1.5.

### Explicit Form of Hamiltonian

In the case of two photonic modes (*N* = 2), the overlap matrix is determined by a single parameter η = η_12_, which determines the expansion coefficient and coupling strength matrix, and the Hamiltonian (eq 2) as follows

11 $$\alpha_{11}=1,,g_{11}=g_{1}$$

12 $$\alpha_{21}=\eta ,,g_{21}=g_{2}\eta$$

13 $$\alpha_{22}=\sqrt{1-\eta^{2}},,g_{22}=g_{2}\sqrt{1-\eta^{2}}$$

$$H=(\begin{array}{llll} E_{ph,1} & 0 & g_{11} & 0\\ 0 & E_{ph,2} & g_{21} & g_{22}\\ g_{11} & g_{21} & E_{ex} & 0\\ 0 & g_{22} & 0 & E_{ex} \end{array})$$ 14

Note that for general *N*, eq 4 must be used to invert the relation between α_*jk*_ and η_*jk*_, with α_*jk*_ = 0 for *k* > *j* and α_11_ real. These constraints can always be applied and reflect the choice of a particular basis for the degenerate exciton states.

### Waveguide Mode Energy Spacing

The energy of waveguide modes *E*_*j*_ is determined by the effective refractive index *n*_eff,*j*_, which depends on the waveguide thickness, the extent of the evanescent field into the surrounding medium, and the mode order. In the limit of negligible evanescent fields, the mode energy can be approximated as a function of the in-plane momentum β = *k*_0_ *n*_eff_ (the propagation constant) as

15 $$E_{j}\left(\beta \right)=\frac{\hbar c}{n_{0}}\sqrt{\beta^{2}+k_{⊥,j}^{2}}$$

where *k*_⊥,*j*_ = *j*π*n*_0_/*D* is the out-of-plane momentum of the *j*-th mode (TE_*j*–1_) due to confinement along the *z*-direction, *k*_0_ is the free-space wavevector, and *n*_0_ is the refractive index of the waveguide material. In the limit where the waveguide is much thicker than the wavelength, a Taylor expansion yields

16 $$E_{j}\left(\beta \right)\approx \frac{\hbar c}{n_{0}}\beta \left[1+\underset{\ll 1}{\underset{︸}{\frac{\pi^{2}n_{0}^{2}}{2\beta^{2}}\frac{j^{2}}{D^{2}}}}\right]$$

## Supplementary Material



## Data Availability

This manuscript was previously made available as a preprint: Johannes Bürger; Erika Cortese; Simone Di Muzio; Daniele Sanvitto; Simone De Liberato; Dario Ballarini. Exciton-Tunable Phase Control and Superstrong Coupling through Multimode Polariton Engineering in Planar Waveguides. 2026, arXiv:2605.27714. arXiv. https://arxiv.org/abs/2605.27714 (accessed September 22, 2026).

## Notes

Overview of works on SSC, details on simulations, additional simulation results for 200, 400, and 600 nm thick waveguides, theoretical model for overlap and coupling strength for ideal waveguide modes, and theoretical model for interference-based mode switching (PDF)
