# Supplementary material for: Exciton-Tunable Phase Control and Superstrong Coupling through Multimode Polariton Engineering in Planar Waveguides
Source: ACS Photonics. 2026 Oct 16;13(21):6373–85. doi: 10.1021/acsphotonics.6c01318 (PMC13741924; doi:10.1021/acsphotonics.6c01318)
Supplement: Supplementary file 1 [file ph6c01318_si_001.pdf]

Supporting Information: Exciton-Tunable Phase Control and Superstrong Coupling Through Multimode Polariton Engineering in Planar Waveguides

Johannes Bürger*[1], Erika Cortese[2], Simone Di Muzio[3], Daniele Sanvitto[1], Simone De Liberato[3], and Dario Ballarini[1]

[1]CNR Nanotec, Institute of Nanotechnology, Via Monteroni, 73100 Lecce, Italy
[2]School of Physics and Astronomy, University of Southampton, Southampton SO17 1BJ, UK
[3]Istituto di Fotonica e Nanotecnologie—Consiglio Nazionale delle Ricerche (CNR), Piazza Leonardo da Vinci 32, Milano, Italy

*Email: johannespascalburger@cnr.it

20 pages | 17 figures | 1 table

Here, we report additional results and relevant background information on the following topics:

Contents

S1 Overview of superstrong coupling regime in different systems **S2**

S2 Optical properties of materials used in the simulation **S3**

S3 Details on simulations and fitting **S3**

S4 Additional simulation results for low-, mid-, and high-thickness waveguides **S3**
S4.1 Low-thickness waveguide (200 nm) . . . S3
S4.2 Mid-thickness waveguide (400 nm) . . . S5
S4.3 High-thickness waveguide (600 nm) . . . S6
Sweep of active material fraction for the high-thickness waveguide . . . S8

S5 Magnitude of energy gap between inner polaritons is not an indicator of mode overlap **S10**

S6 Theoretical considerations on optimizing the mode overlap **S11**
S6.1 Overlap and coupling strength optimization in ideal waveguides . . . S11
S6.2 Overlap and coupling strength optimization for higher-order modes . . . S15

S7 Interference-based mode switching **S17**

S1 Overview of superstrong coupling regime in different systems

Energy	Temp.	Matter excitation	Photonic components	Mode orders	Mode spacing $\Delta E_{\mathrm{ph},jk}$	Coupling g_j	SSC ratio $g_j/\Delta E_{\mathrm{ph},jk}$	Active fraction	Paper analysis includes...	Ref.
VIS (517 nm)	RT	Perovskite exciton	Planar waveguide<br>(200 nm thick)<br>(400 nm thick)<br>(600 nm thick)	1-2	391 meV<br>152 meV<br>80 meV	44-49 meV<br>40-58 meV<br>37-59 meV	0.11-0.13<br>0.26-0.38<br>0.46-0.74	30%	Overlap ✓, Hopfield coefficients ✓<br>SSC ✓, S-shape in theory ✓	2026 This work (theory)
VIS (606 nm)	RT	Perovskite exciton	Tunable cavity	∼5-6	∼360 meV	78 meV	0.22	∼15%	Overlap ✗, Hopfield coefficients ✗<br>SSC ✓, S-shape in theory ✓	2025 [1]
VIS (605 nm)	RT	Perovskite exciton	Microcavity	6-8	-	-	-	18%	Overlap ✓, Hopfield coefficients ✗<br>SSC ✗, S-shape ✓	2023 [2]
VIS (588 nm)	RT	J-aggregate dye exciton	Microcavity	11-12	190 meV	48 meV	0.25	∼15%	Overlap ✗, Hopfield coefficients ✓<br>SSC ✓, S-shape only for low g_j ✓	2021 [3]
VIS (590 nm)	RT	J-aggregate dye exciton	Microcavity	3-4<br>8-9	∼600 meV<br>∼200 meV	64 meV<br>64 meV	0.11<br>0.32	100%<br>100%	Hopfield coefficients ✓<br>SSC theory (100% active fraction) ✓<br>S-shape only for low g_j ✓	2023 [4]
NIR (852 nm)	Cold atoms	Cs ensemble	Fiber ring resonator	$\sim 5 \times 10^7$	7.1 MHz	8.7 MHz	1.23	low	Overlap ✗, Hopfield coefficients ✗<br>SSC ✓, S-shape ✗	2019 [5]
THz (360 GHz)	Cryo	GaAs QWs cyclotron resonance	3D photonic crystal cavity	1-2	∼44 GHz	∼72 GHz	1.64	0.09 %	Overlap ✓, Intermode correlations ✓<br>USC+SSC ✓, S-shape ✓	2025 [6]
THz (1.2 THz)	Cryo	GaAs QWs cyclotron resonance	patterned metasurface	1-2	0.8 THz	0.22-0.43 THz	0.28-0.54	low	Overlap ✓, Hopfield coefficients ✗<br>USC+SSC ✓, S-shape ✓	2024 [7]
Microwave (5-13 GHz)	Cryo	Transmon qubit	Superconducting cavity	∼ 250	∼50 MHz	∼65 MHz	1.30	low	Overlap ✗, Hopfield coefficients ✗<br>SSC ✓, S-shape (very clean) ✓	2019 [8]
Microwave (10 GHz)	Cryo	Superconducting resonator	Acoustic resonator	∼1000	9.2 MHz	2.4 MHz	0.26	-	Overlap ✗, Hopfield coefficients ✗<br>SSC ✓, S-shape ✓	2016 [9]

Table S1: Summary of selected experimental works on superstrong coupling compared to our work. "Active fraction" is the ratio of active material volume to total mode volume. If references did not report certain values they were estimated from their data (marked by ∼) or omitted (marked "-"). Aspects analyzed in the papers are classified as: ✓ = included in paper, ✓ = partially included ✗ = not included. RT: room temperature, Cryo: cryogenic temperatures.

S2 Optical properties of materials used in the simulation

The permittivity $\epsilon(\omega)$ of the active material is described by a Lorentzian model:

$$\epsilon(\omega) = \epsilon_\infty + \frac{\omega_p^2}{\omega_0^2 - \omega^2 - \mathrm{i}\gamma_{\mathrm{ex}}\omega} \tag{1}$$

As reported in the main text, the parameters used in the simulations are:

$\epsilon_\infty = 2.3^2 \qquad \hbar\omega_0 = 2.398$ eV $\qquad \hbar\omega_{\mathrm{p}} = 0.5$ eV $\qquad \hbar\gamma_{\mathrm{ex}} = 30$ meV

With this definition, γ_{ex} corresponds to the full width at half maximum (FWHM) of $\mathrm{Im}(\epsilon)$. The dielectric filler is assumed to be lossless, with its permittivity matched to the background value of the active material ϵ_∞. The substrate is assumed to be a semi-infinite lossless SiO_2 layer with a constant refractive index of 1.5.

S3 Details on simulations and fitting

The excitation is a plane wave incident at an angle θ_{inc} with respect to the surface normal (z axis) of the waveguide. The incident wavevector lies in the xz plane, resulting in the electric field being polarized along the y axis. During the testing phase, 20 Fourier orders were used, while 40 were used for the final plots included in this publication. The reported reflectance values are defined as the ratio of reflected to incident power.

The corresponding parameter settings in S^4 are (where θ_{inc} corresponds to `phi` in the S^4 naming convention):

```
IncidenceAngles = (phi, 0)
sAmplitude = 1
pAmplitude = 0
NumBasis = 40
```

To fit the theoretical model, reflection dips or peaks are identified from the second derivative of the reflectance spectrum with respect to energy.

The dispersion of the uncoupled photonic modes is extracted by setting the oscillator strength of the exciton to zero ($\omega_{\mathrm{p}} = 0$). Within the studied energy and incidence angle range, the resulting dispersion curves are accurately described by a second-order polynomial, obtained by fitting $E_{\mathrm{ph},j}(\theta_{\mathrm{inc}})$.

The extracted fit parameters are used to construct the photonic part of the Hamiltonian in Equation (2) of the main text. This Hamiltonian is then fitted to the polariton dispersion from the full simulation (with nonzero oscillator strength) by minimizing the RMS error between the fit and simulated data. The fitting parameters are the coupling strengths g_j and the overlap matrix η_{jk} between the photonic modes.

S4 Additional simulation results for low-, mid-, and high-thickness waveguides

For completeness, we provide the full fitting results that were discussed in the main text here.

S4.1 Low-thickness waveguide (200 nm)

We note that for this specific geometry, the overlap coefficient η_{12} could not be reliably extracted from the fit, as the RMS error depends only weakly on the overlap value. The fitting procedure yields an overlap value of $\eta_{12} = 0.883$ but leaves a large gap between the polaritons that is not present in the simulated data. This issue could be resolved by giving more weight to the data points near the crossover region.

Instead, here we resort to manually fixing the overlap value to its expected value for a 30% layer at the bottom of the waveguide, which is $\eta_{12} = 0.99$. We justify this by noting that all values of $\eta_{12} < 0.97$ leave a significant gap between the modes, which is absent in the simulated data and can therefore be excluded.

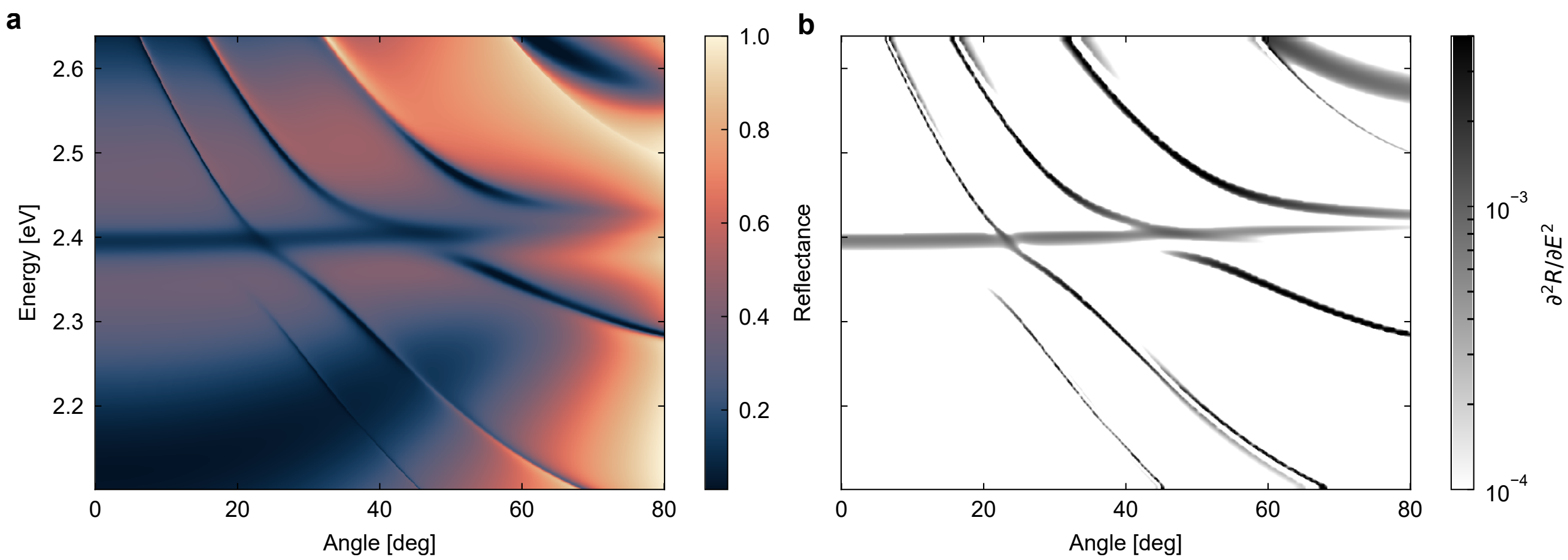

Figure S1: Comparison between reflectance spectrum and its second derivative for the mid-thickness waveguide (400 nm) presented in Figure 3. (a) Original reflectance spectrum. (b) Second derivative of the reflectance spectrum. The dips in the reflectance spectrum correspond to positive peaks in the second derivative. Negative values of the second derivative are rendered as white for clarity. Note that the narrow mode visible on the top right at (2.5 eV, 80°) is not a waveguide mode and does not couple significantly to the exciton. It is therefore omitted from the analysis.

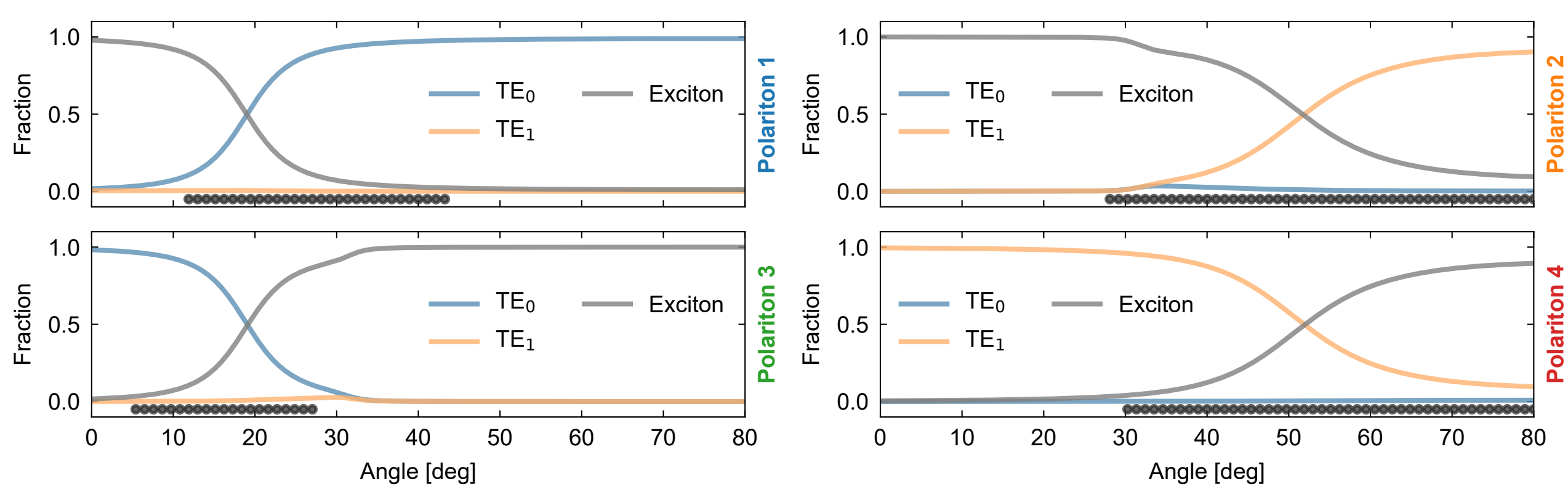

Figure S2: Fractions of uncoupled modes in the 4 polaritons of the thin waveguide (200 nm) shown in Figure 2, as obtained from the fit. The crossover from TE_0 to TE_1 occurs between polaritons 2 and 3. Gray dots mark the angle values used for fitting each polariton. Note that the presence of dots indicates that the reflection contrast was sufficient to resolve the mode at the corresponding angle. The absence of dots may arise from insufficient contrast, the mode extending beyond the investigated energy interval, or because points where the mode is nearly purely excitonic were not included in the fit.

S4.2 Mid-thickness waveguide (400 nm)

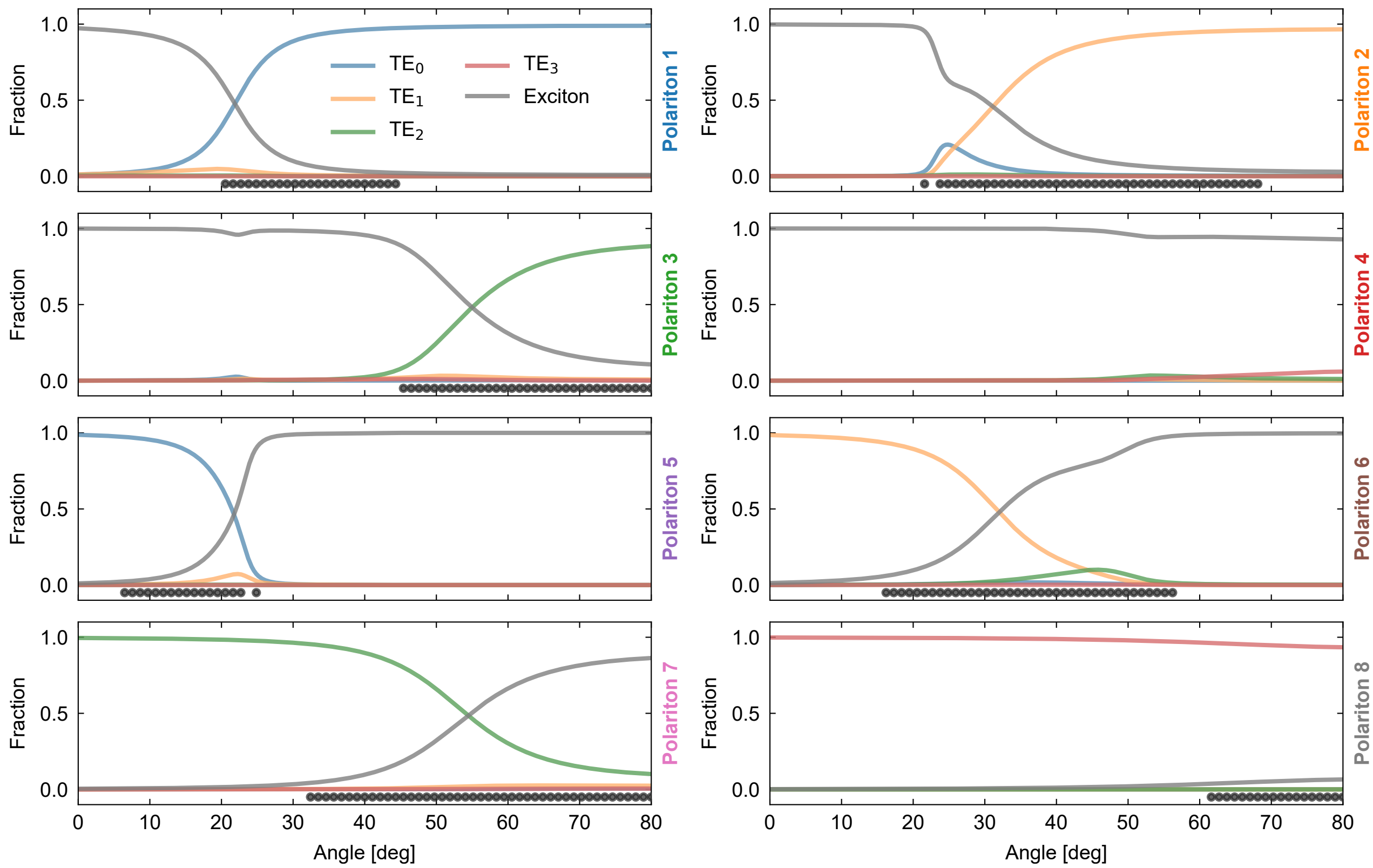

Figure S3: Fractions of uncoupled modes in the 8 polaritons of the mid-thickness waveguide (400 nm) shown in Figure 3, as obtained from the fit. The crossover from TE_0 to TE_1 occurs between polaritons 5 and 2. Gray dots mark the angle values used for fitting each polariton. Note that the presence of dots indicates that the reflection contrast was sufficient to resolve the mode at the corresponding angle. The absence of dots may arise from insufficient contrast, the mode extending beyond the investigated energy interval, or because points where the mode is nearly purely excitonic were not included in the fit.

S4.3 High-thickness waveguide (600 nm)

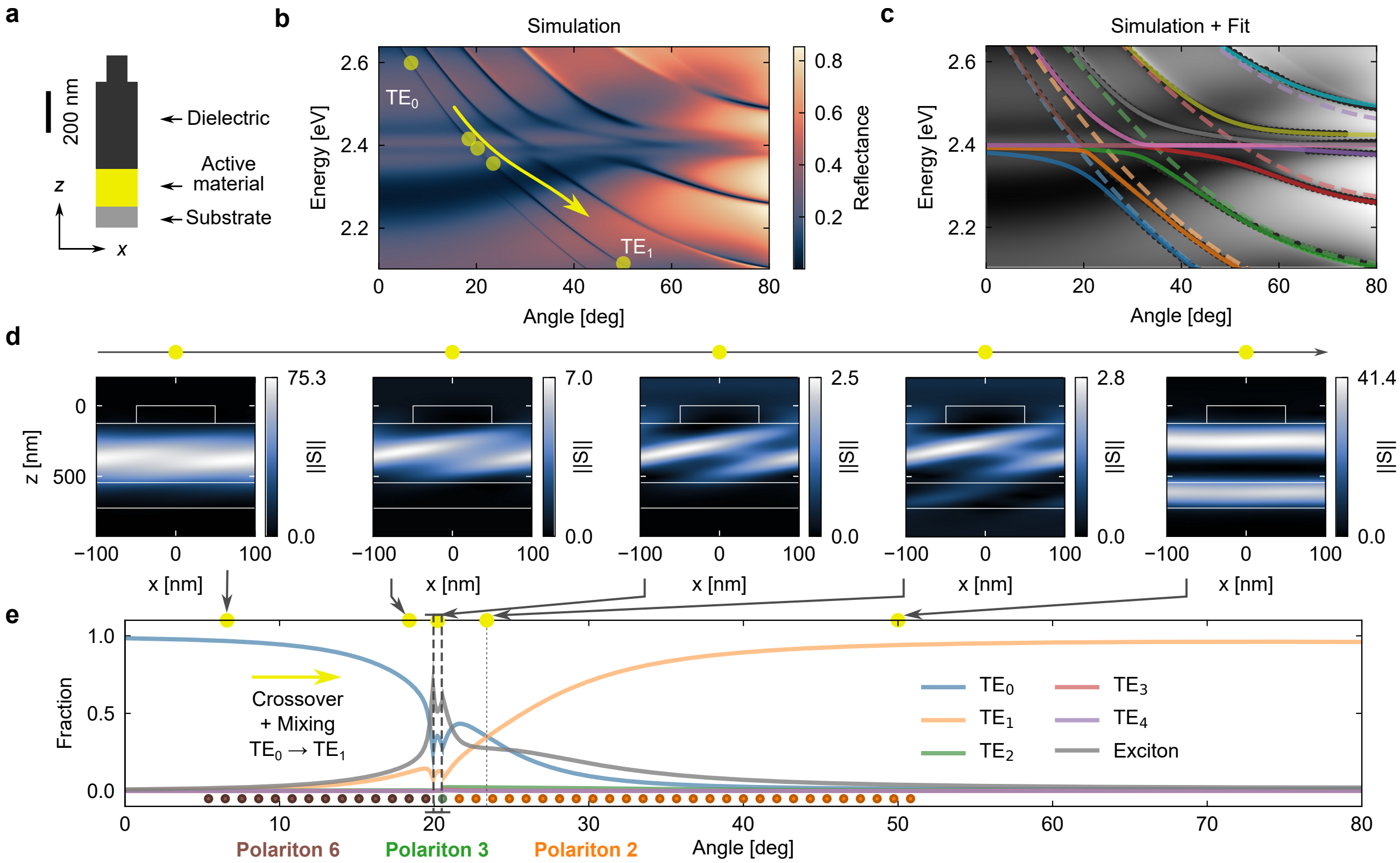

Figure S4: Simulation of superstrong coupling in the high-thickness waveguide ($D = 600$ nm). (a) Unit cell of the grating structure with active material fraction $\Delta z/D = 0.3$ and periodicity $P = 200$ nm. (b) Polariton dispersion in reflection. (c) Fit using TE_0-TE_4 photonic modes (dashed lines), yielding 10 polariton branches. Gray dots indicate points used in fit. (d) Poynting vector magnitude at selected yellow points in (b), illustrating the crossover from TE_0 to TE_1. (e) Fractions of uncoupled modes in polaritons 2 (orange, right), 3 (green, middle), and 6 (brown, left) separated by the crossover lines (gray dashed). No significant discontinuity is observed due to the small energy gap. Colored dots mark angles used in the fit, their presence indicates sufficient reflection contrast.

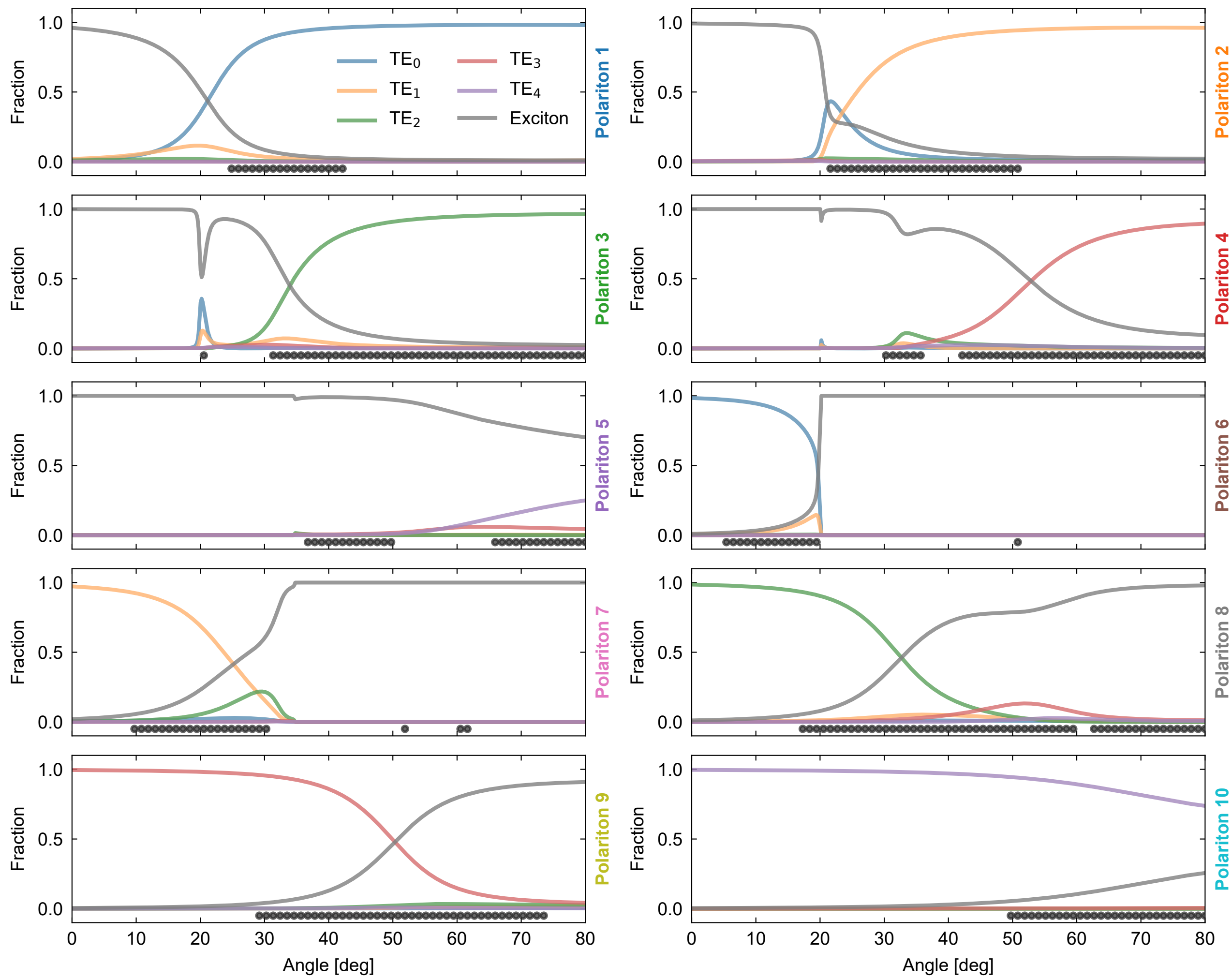

Figure S5: Fractions of uncoupled modes in the 10 polaritons of the high-thickness waveguide (600 nm) shown in Figure S4, as obtained from the fit. The crossover from TE_0 to TE_1 occurs between polaritons 6, 3 and 2. Gray dots mark the angle values used for fitting each polariton. Note that the presence of dots indicates that the reflection contrast was sufficient to resolve the mode at the corresponding angle. The absence of dots may arise from insufficient contrast, the mode extending beyond the investigated energy interval, or because points where the mode is nearly purely excitonic were not included in the fit.

Sweep of active material fraction for the high-thickness waveguide

The parameters for the grating structure are the same as in Figure S4.

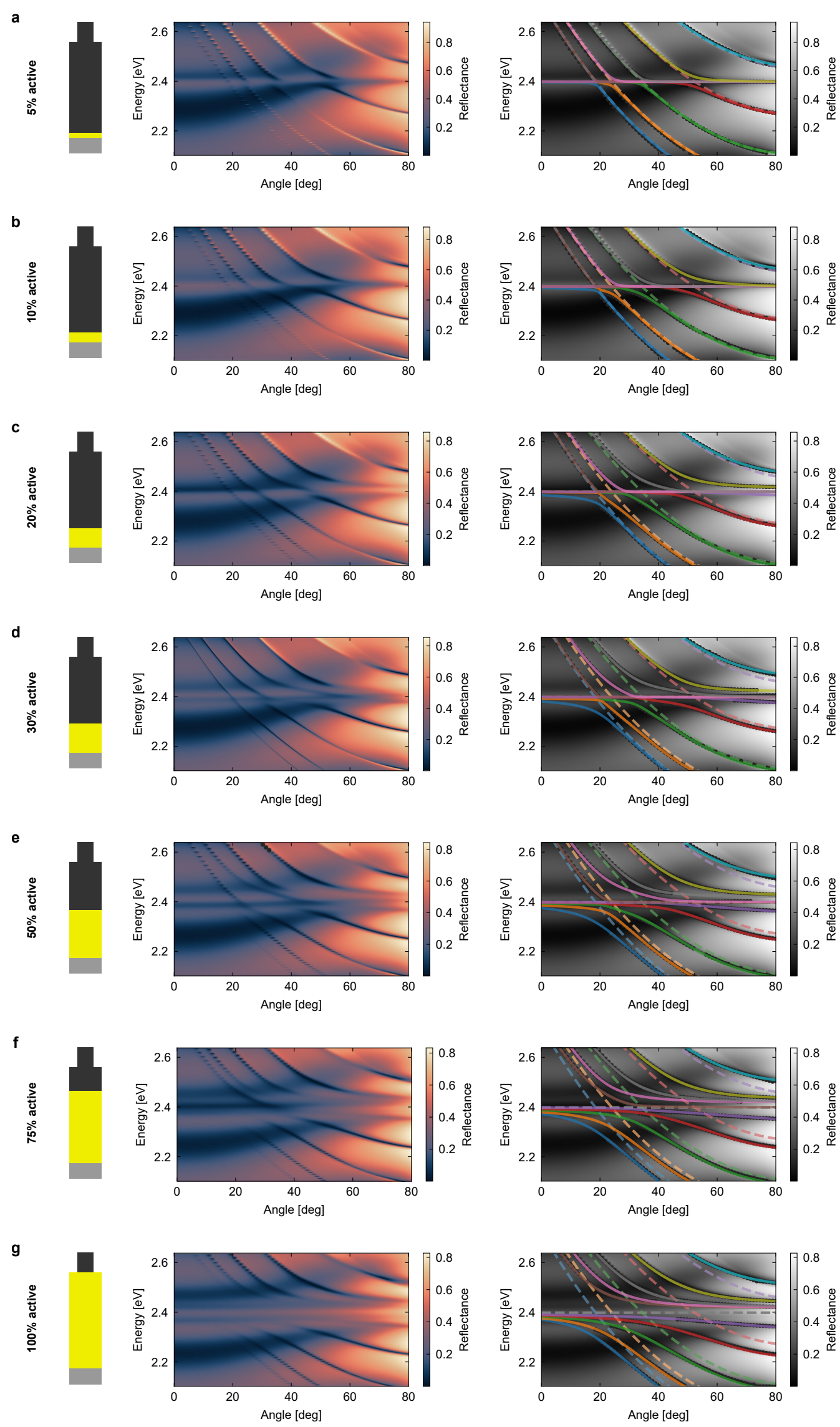

Figure S6: Simulation of high-thickness waveguide (D = 600 nm) for different active material fractions $\Delta z/D$: 5% (a), 10% (b), 20% (c), 30% (d), 50% (e), 75% (f), and 100% (g). Left: Geometry of the waveguide. Middle: Polariton dispersion in reflection. Right: Fit using TE_0-TE_4 photonic modes (dashed lines), yielding 10 polariton branches. Gray dots indicate points used in the fit.

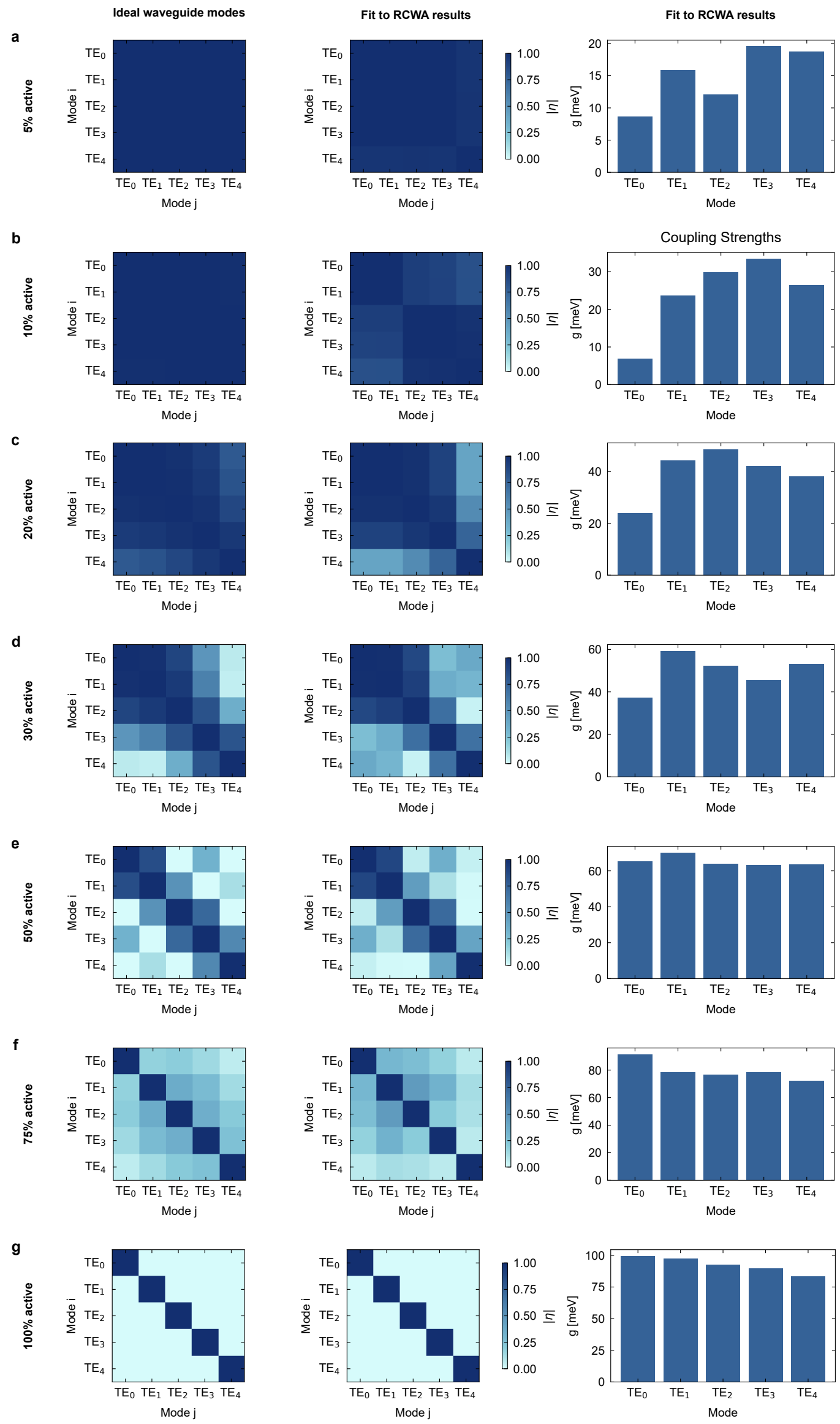

Figure S7: Continuation of Figure S6: Simulation of high-thickness waveguide (D = 600 nm) for different active material fractions $\Delta z/D$: 5% (a), 10% (b), 20% (c), 30% (d), 50% (e), 75% (f), and 100% (g). Left: Overlap matrix η_{jk} obtained from Equation (6). Middle: Overlap matrix η_{jk} obtained from the fits shown in Figure S6. Right: Total coupling strengths g_j obtained from the fit.

S5 Magnitude of energy gap between inner polaritons is not an indicator of mode overlap

In the general case of an imperfect overlap, $\eta_{jk} < 1$, the used Hamiltonian (Equation (2)) of the main text predicts a finite energy gap $\Delta E_{\mathrm{pol},jk}$ between the two inner polaritons at the crossover. If this energy gap is smaller than the linewidth of the polaritons, the gap cannot be resolved and the transition appears continuous.

One might expect the magnitude of this energy gap to be a direct indicator of the mode overlap, with smaller gaps corresponding to higher overlaps. However, this is not the case, as the gap size also depends on the coupling strength. A small gap can result from either a high overlap or a low coupling strength. In fact, there are infinitely many combinations of overlap and coupling strength that yield the same gap size, as illustrated by the ochre contour lines in Figure S8(c). Therefore, the mode overlap cannot be reliably inferred from the gap size alone and must instead be extracted from a full fit of the polariton dispersion.

Note that the Hamiltonian used here does not explicitly include losses as parameters. The presence or absence of a continuous crossover in the dispersions can therefore only be inferred by comparing the calculated gap size to the linewidth of the polaritons at the crossover. A different model that explicitly includes losses but is restricted to the limiting cases of zero or unity overlap, has been developed for microcavities [10].

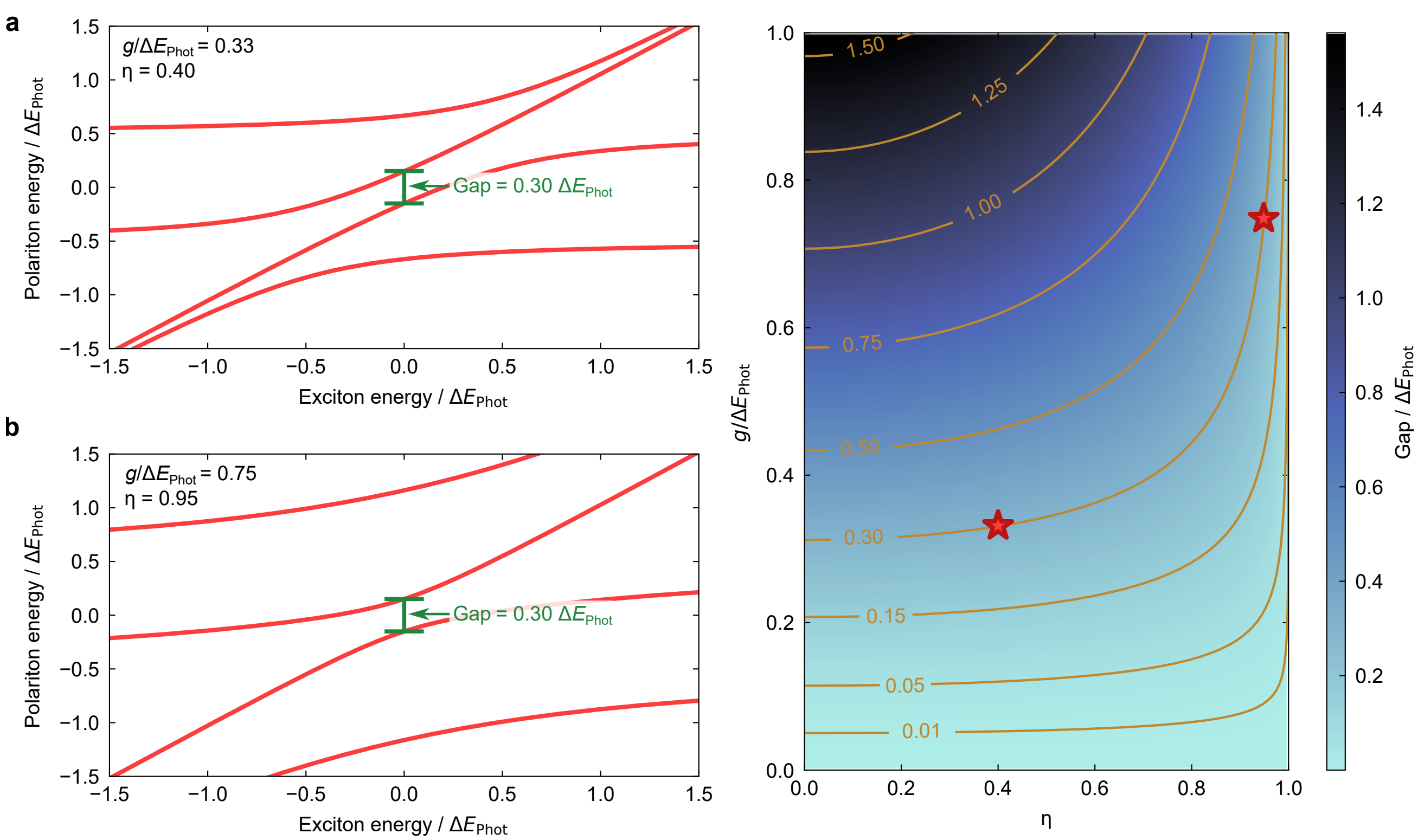

Figure S8: Energy gap between inner polaritons as a function of coupling strength and mode overlap, calculated using the Hamiltonian in Equation (2) of the main text for the case of two photonic modes. (a) Polariton energies for varying exciton energies for a combination of low overlap and low coupling strength. (b) Polariton energies for a combination of high overlap and high coupling strength yielding the same energy gap (green marker) as in (a). (c) Energy gap between the inner polaritons as a function of coupling strength and mode overlap. The ochre contour lines indicate the combinations of coupling strength and overlap that yield a given gap size. All energies are normalized to the photonic mode separation $\Delta E_{\mathrm{ph},12}$, and the photonic modes are assumed to have energies of $\pm\Delta E_{\mathrm{ph},12}/2$.

S6 Theoretical considerations on optimizing the mode overlap

In order for the involved photonic modes to couple efficiently, their overlap η_{jk} needs to be optimized which is achieved by strategically placing the active material where the modal overlap is high.

We start by assuming that the electric field profiles $F_{y,\mathrm{TE}_0}(x,z)$ and $F_{y,\mathrm{TE}_1}(x,z)$ of the two photonic modes in the presence of the active material are known. (The y dependence is omitted due to the invariance of the structure in this direction.) In practice, we use the waveguide modes in the absence of the active material as an initial guess, which could later be refined iteratively once the first round of the optimization is complete.

In the case of the layered fabrication approach that we target in this work, the most general form for the overlap given a certain desired total thickness of the active material Δz arranged in a number n_{layer} of layers is:

$$\eta_{12} = \frac{\sum_{i=1}^{n_{\mathrm{layer}}} \int_{a_i}^{b_i} dz \int dx \, F^*_{y,\mathrm{TE}_0}(x,z) F_{y,\mathrm{TE}_1}(x,z)}{\sqrt{\left(\sum_{i=1}^{n_{\mathrm{layer}}} \int_{a_i}^{b_i} dz \int dx \, |F_{y,\mathrm{TE}_0}(x,z)|^2\right) \left(\sum_{i=1}^{n_{\mathrm{layer}}} \int_{a_i}^{b_i} dz \int dx \, |F_{y,\mathrm{TE}_1}(x,z)|^2\right)}}, \tag{2}$$

where a_i, b_i describe the boundaries of layer i along the z direction, which are constrained by $\Delta z = \sum_{i=1}^{n_{\mathrm{layer}}} b_i - a_i$, and the integral over dx runs over the complete length of the unit cell of the grating.

The optimization procedure in this general case would encompass finding the optimal values of a_i, b_i and the number of layers n_{layer} in order to maximize η_{12}. This is a numerically demanding task, the solution of which might benefit from techniques used in generalizations of the maximum subarray problem.

In our work, we are restricting ourselves to $n_{\mathrm{layer}} = 1$ as higher numbers of layers would only become beneficial when targeting the coupling of modes of higher order, or when the grating strongly distorts the waveguide modes. In the case of $n_{\mathrm{layer}} = 1$, the optimization procedure is simplified to maximizing:

$$\eta_{12}(z_0) = \frac{\int_{z_0-\Delta z/2}^{z_0+\Delta z/2} dz \int dx \, F^*_{y,\mathrm{TE}_0}(x,z) F_{y,\mathrm{TE}_1}(x,z)}{\sqrt{\left(\int_{z_0-\Delta z/2}^{z_0+\Delta z/2} dz \int dx \, |F_{y,\mathrm{TE}_0}(x,z)|^2\right) \left(\int_{z_0-\Delta z/2}^{z_0+\Delta z/2} dz \int dx \, |F_{y,\mathrm{TE}_1}(x,z)|^2\right)}}, \tag{3}$$

as a function of the center position z_0 of the active material layer, given a fixed thickness Δz. Note that due to the terms in the denominator, this optimization procedure is different from just picking the locations where the product $F_{y,\mathrm{TE}_0}(x,z)F_{y,\mathrm{TE}_1}(x,z)$ is maximal.

In Section S6.1, we show the analytical solution for this optimization problem for an ideal waveguide. For the simulation results in the main text, this problem is solved numerically with a brute-force approach.

S6.1 Overlap and coupling strength optimization in ideal waveguides

As we will see, a high overlap can quite easily be achieved by using infinitesimally thin layers of the active material (e.g., TMD monolayers). However, this comes at the cost of low coupling strength as the fraction of the mode volume filled by the active material would be small. To outline this tradeoff between overlap η_{12} and coupling strengths g_1 and g_2 between the photonic modes and the exciton in the active material, we study the problem on the example of an ideal waveguide.

To this end, we assume perfectly reflecting waveguide walls and a negligibly thin grating, resulting in the following simple electric field profiles (corresponding to Equation (6) of the main text):

$$\begin{aligned} F_{y,\mathrm{TE}_0}(x,z) &= \sin\left(\frac{\pi z}{D}\right), \\ F_{y,\mathrm{TE}_1}(x,z) &= \sin\left(\frac{2\pi z}{D}\right) \end{aligned} \tag{4}$$

where D is the total thickness of the waveguide (Figure S9(a)). In this case, the integrals in Equation (3) and the maximization of η_{12} can in principle be solved analytically. However, the resulting expressions are lengthy and not particularly instructive. Therefore, we resort to numerical methods.

First, we show the dependence of the overlap on the position of the active material for a few different thicknesses Δz of the active material (Figure S9(b)).

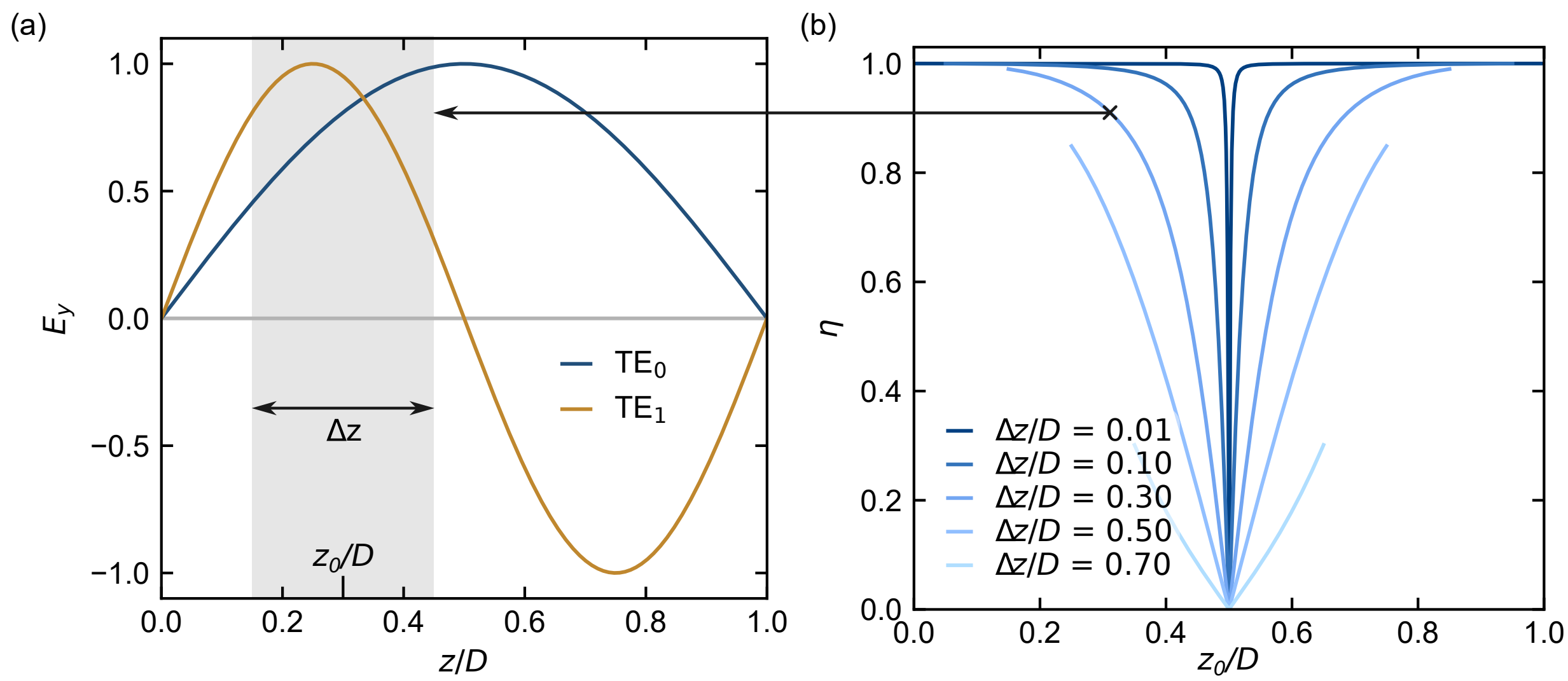

Figure S9: Dependence of mode overlap on position of active material in an ideal waveguide. (a) Electric field of ideal TE_0 and TE_1 waveguide modes. Gray rectangle indicates an example placement of the active material with center position z_0 and thickness Δz in a waveguide of total thickness D (b) Mode overlap as a function of the active material position for several values of its thickness. Curves terminate where the thickness of the material restricts the allowable positions.

As evident, the highest overlap attainable for any given thickness is obtained when the active material is placed at either the top- or bottommost position within the waveguide (maximal or minimal allowed value of z_0). In the limit of an infinitesimally thin active layer located at these positions, the overlap approaches unity. This behavior can be understood by noting that at the waveguide boundaries ($z/D = 0$ or $z/D = 1$), both electric fields vanish and may be approximated locally by the linear term of their respective Fourier expansions, albeit with different slopes. Upon normalization according to Equation (3), these linear field profiles become identical, and as such yield an overlap of 1 by definition.

In general, thinner materials achieve a high overlap in any location of the waveguide, except in the center where positive and negative contributions to the overlap integral compensate each other. With increasing thickness of the material, the maximally achievable overlap decreases.

To illustrate this point further, we numerically calculate the maxima of Equation (3) for a given thickness of the active material, yielding the highest achievable overlap and the optimal position of the material (Figure S10). The result of the optimization confirms that the optimal position for the active material to achieve high overlap is the top- or bottommost position within the waveguide.

To quantify how strongly each photonic mode interacts with the active material in this case, we use the following relative measure of the coupling strength [11]:

$$g_{\text{rel}} = \sqrt{\int_{z_0-\Delta z/2}^{z_0+\Delta z/2} dz\, |F_y|^2} \tag{5}$$

With this definition, we can rewrite Equation (3) as:

$$\eta(z_0) = \frac{\int_{z_0-\Delta z/2}^{z_0+\Delta z/2} dz \int dx\, F^*_{y,\text{TE}_0}(x,z) F_{y,\text{TE}_1}(x,z)}{g_{\text{rel},\text{TE}_0}\; g_{\text{rel},\text{TE}_1}}. \tag{6}$$

As the relative coupling strengths appear in the denominator, Equation (6) directly shows the tradeoff between coupling strength and overlap. This is evident in Figure S10(b), where the coupling strengths decrease as the optimal solution achieves higher overlap.

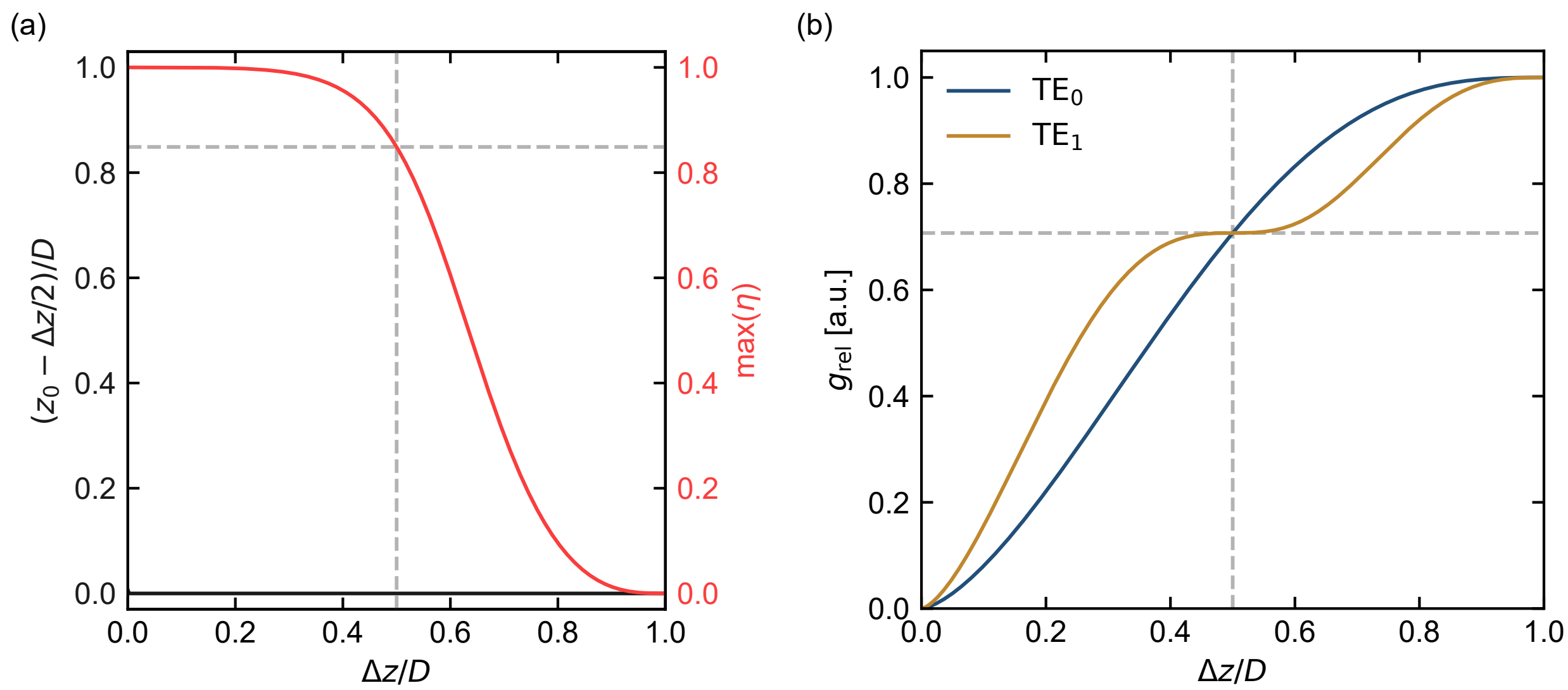

Figure S10: 1D Optimization of the overlap η_{12} for a given material thickness Δz using ideal TE_0 and TE_1 waveguide modes. (a) Maximally achievable overlap and the corresponding optimal position of the material. The result of the optimization places the material at the bottom of the waveguide for all thicknesses. By symmetry, an equivalent solution exists at the top (position not shown for clarity). (b) Relative coupling strengths corresponding to the solutions in (a). $g_{\mathrm{rel}} = 1$ corresponds to the coupling strength in a completely filled waveguide.

As a practical result of this discussion, when the lower (or upper) half of the waveguide is filled by the active material ($\Delta z = 0.5$) one can expect an overlap of the TE_0 and TE_1 modes of $\eta_{12} = 0.85$ and an equal coupling strength for both modes that reaches $1/\sqrt{2}$ of the value in the completely filled waveguide.

Although the previous optimization provided clear guidance, one might still wonder whether a better solution exists that directly maximizes the coupling strength for a given overlap, treating both z_0 and Δz as free optimization parameters. To address this, we performed a 2D brute-force optimization using the geometric mean of the coupling strengths $\sqrt{g_{\mathrm{rel,TE_0}}\ g_{\mathrm{rel,TE_1}}}$ as the optimization target (Figures S11 and S12). Again, we find that the optimal configuration places the active material at either the top- or bottommost position of the waveguide. The results in Figure S11 therefore provide a useful guideline for the design of realistic geometries, illustrating the tradeoff between coupling strength and mode overlap.

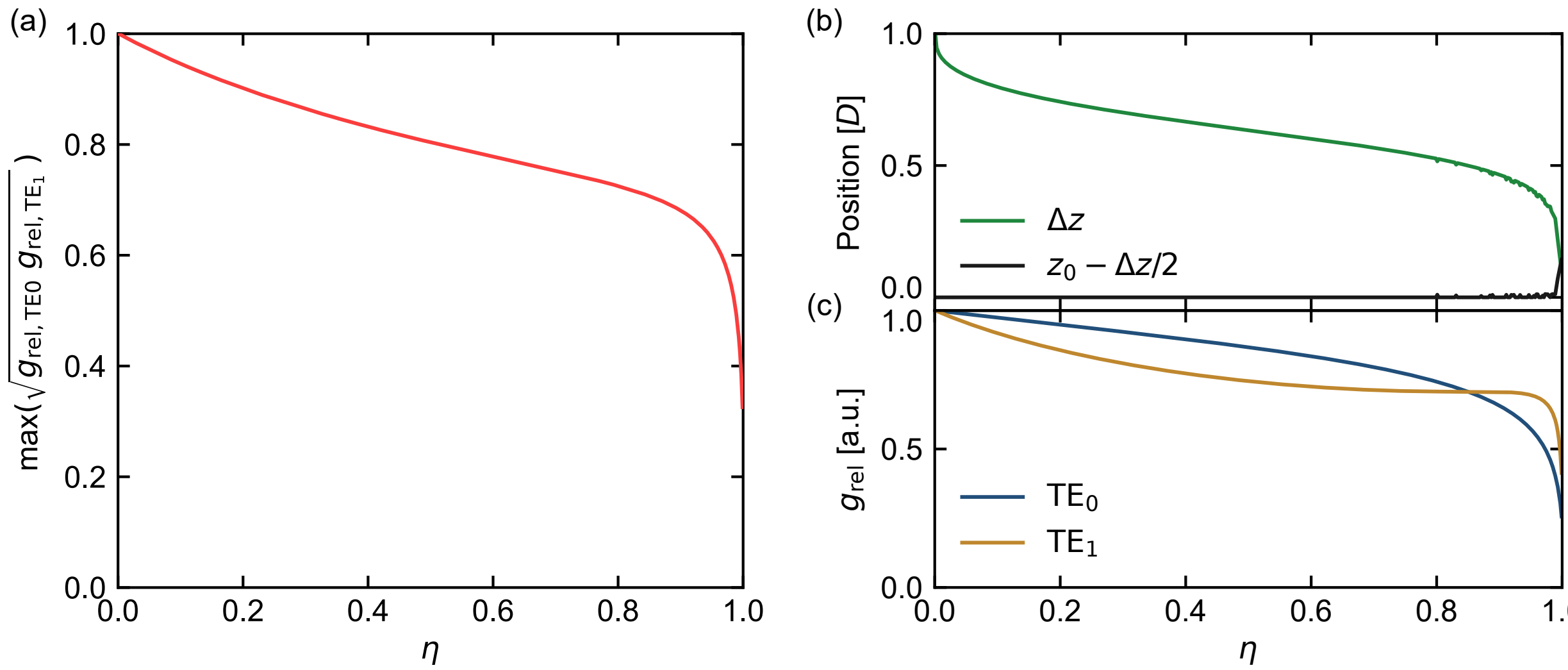

Figure S11: 2D Optimization of the geometric mean of the coupling strengths for a given overlap η_{12} using ideal TE_0 and TE_1 waveguide modes, with both material thickness Δz and position z_0 as free parameters. (a) Maximally achievable geometric mean of the coupling strengths for a given overlap. (b) The optimal values of the position and thickness of the active material to achieve the solution in (a). The result of the optimization places the material at the bottom of the waveguide for all thicknesses. By symmetry, an equivalent solution exists at the top (position not shown for clarity). (c) The resulting individual coupling strengths for the TE_0 and TE_1 modes for the solution in (a). $g_{rel} = 1$ corresponds to the coupling strength in a completely filled waveguide. For large η_{12}, the low thickness causes numerical noise due to many nearly equivalent positions (cf. Figure S9(b)).

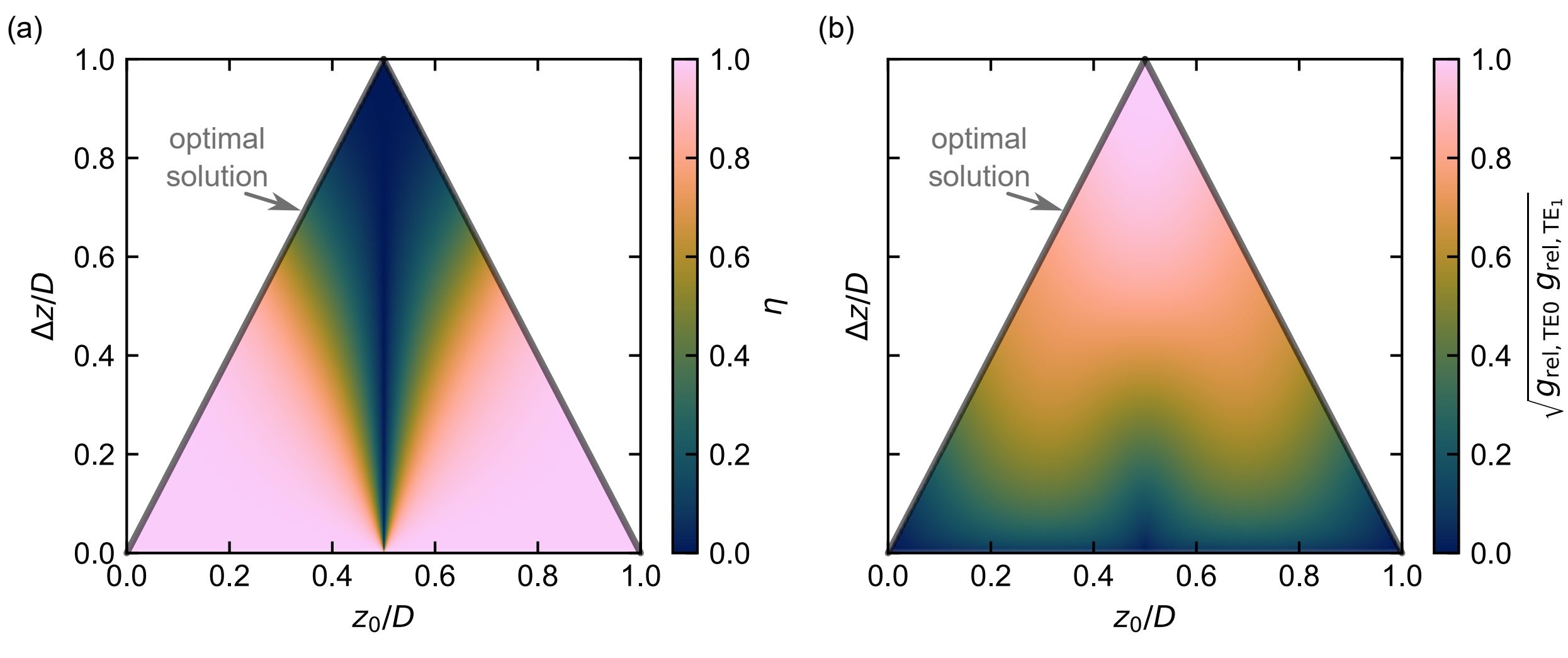

Figure S12: Dependence of (a) the overlap η_{12} and (b) the geometric mean of the coupling strengths on the material thickness Δz and position z_0 for ideal TE_0 and TE_1 waveguide modes. This library is used for the brute-force optimization shown in Figure S11. The optimal solution, corresponding to placement of the active material at the top- or bottommost position of the waveguide, is highlighted by the gray line.

S6.2 Overlap and coupling strength optimization for higher-order modes

Although we are focusing on the TE_0 and TE_1 modes in this manuscript, we are including the situation that the simple model predicts for the TE_3 and TE_4 modes of the ideal waveguide, analogous to the treatment used before.

As shown in Figures S13 and S14, when the thickness of the active material is fixed (e.g., due to experimental constraints) the position yielding the highest overlap does not generally coincide with the top- or bottommost placement. Moreover, the overlap does not increase monotonically with decreasing thickness for higher-order modes, making certain thickness values unfavorable.

By contrast, when the thickness is allowed to vary freely, as in the 2D optimization of the geometric mean of the coupling strengths at a fixed overlap (Figure S15), the optimal solution consistently places the active material at the top- or bottommost position. The results in Figure S15 now exhibit discontinuities at points where a further reduction in thickness no longer leads to an increase in overlap, thus requiring an abrupt change in the optimal thickness.

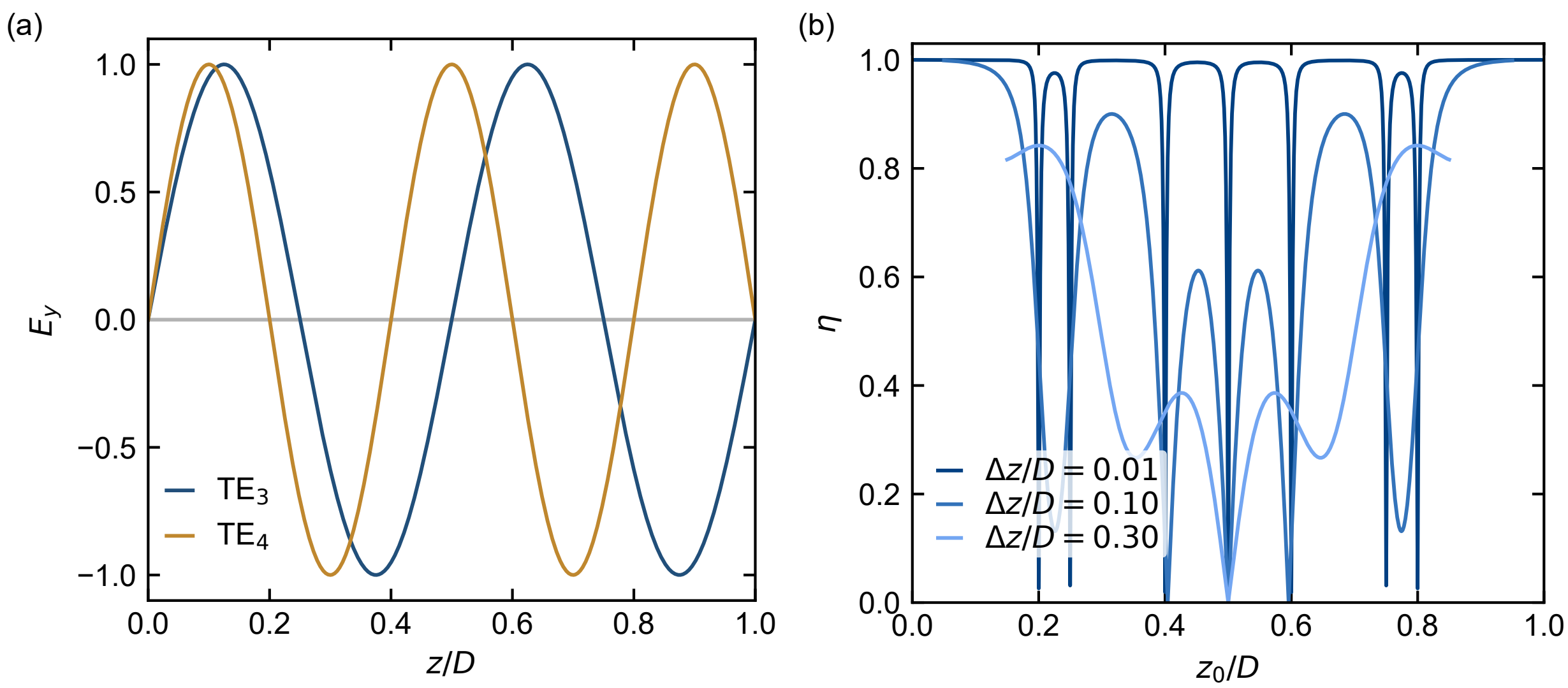

Figure S13: Dependence of mode overlap on position of active material for higher-order modes. (a) Electric field of ideal TE_3 and TE_4 waveguide modes. (b) Mode overlap as a function of the active material position for several values of its thickness. Curves terminate where the thickness of the material restricts the allowable positions. For thin active layers, the overlap exhibits 7 minima at positions where one of the modes crosses zero while the other remains finite.

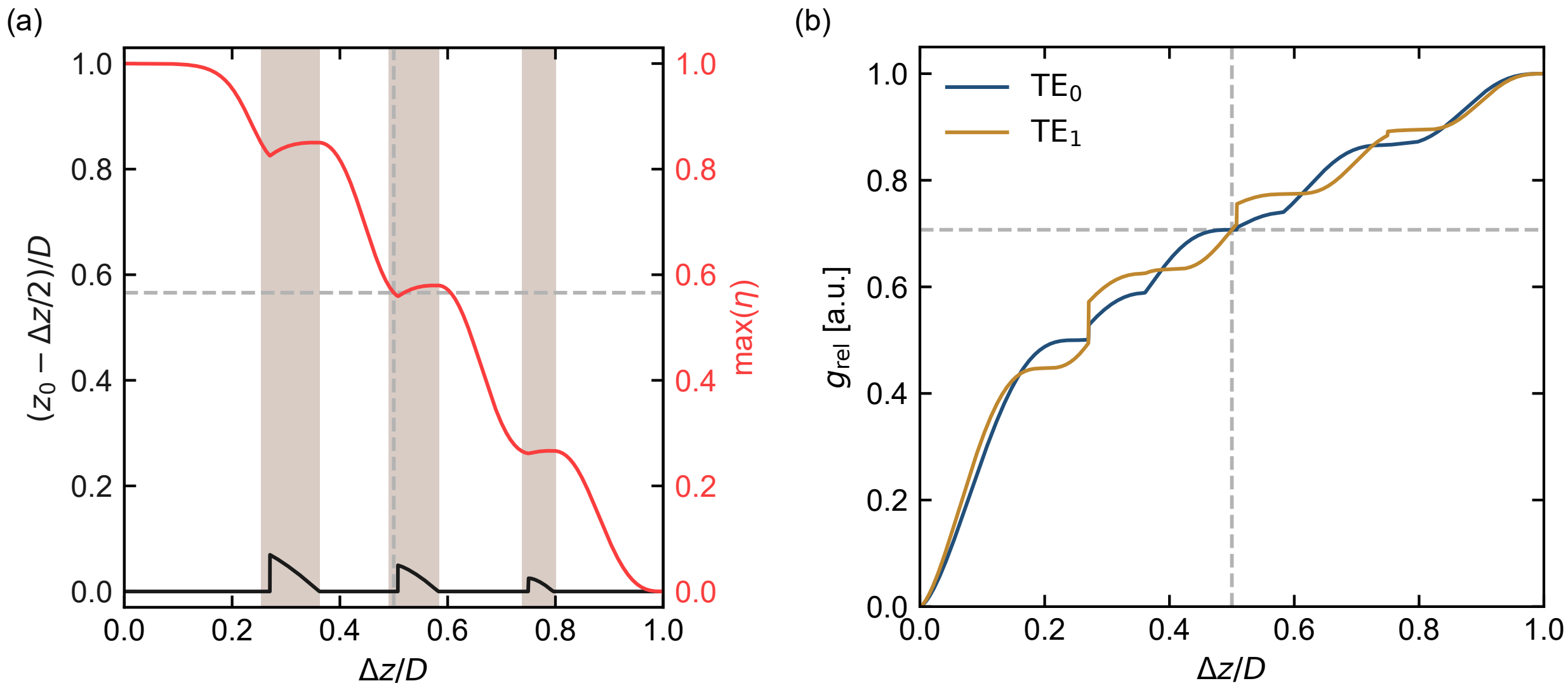

Figure S14: 1D Optimization of the overlap η_{45} for a given material thickness Δz using ideal TE_3 and TE_4 waveguide modes. (a) Maximally achievable overlap and the corresponding optimal position of the material. Brown shading highlights where overlap does not increase with decreasing material thickness. By symmetry, an equivalent solution exists at the top (position not shown for clarity). (b) Relative coupling strengths corresponding to the solutions in (a). $g_{\rm rel} = 1$ corresponds to the coupling strength in a completely filled waveguide.

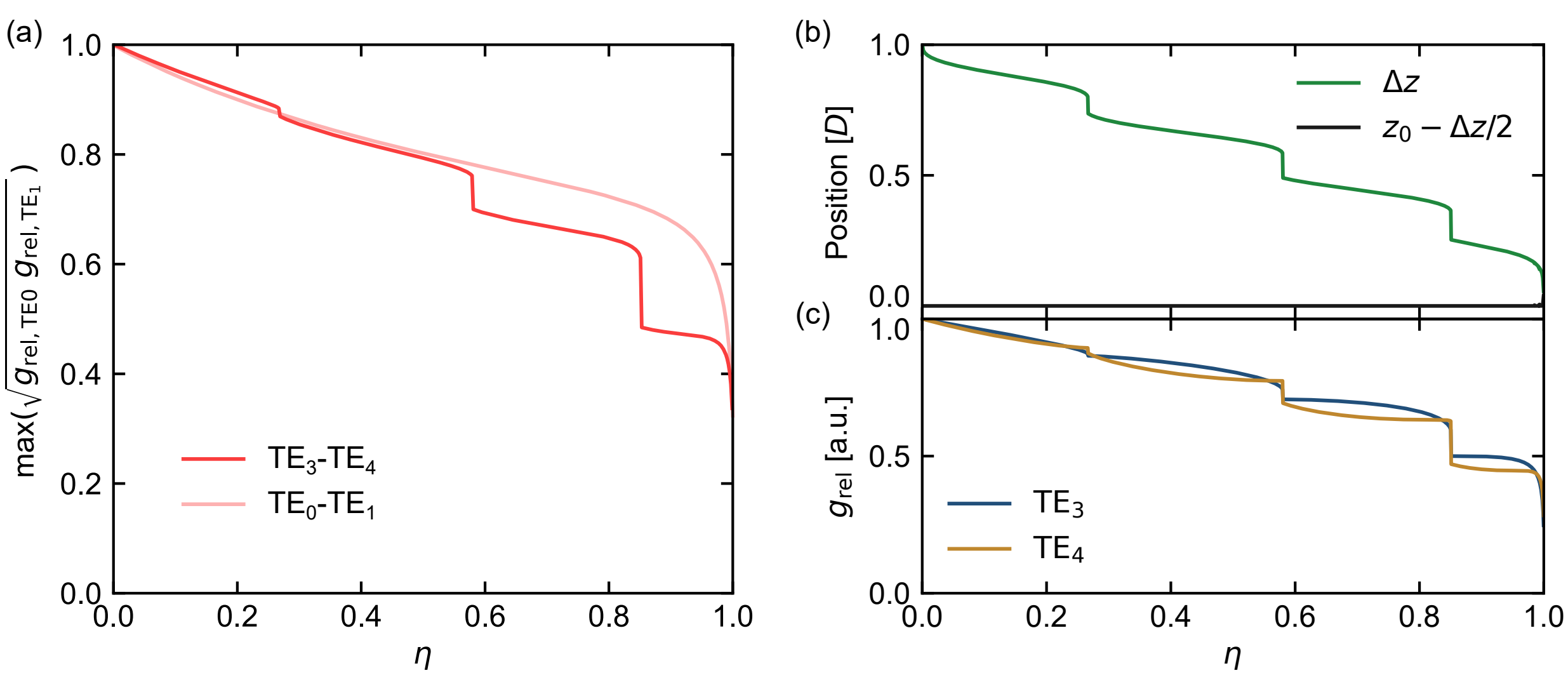

Figure S15: 2D Optimization of the geometric mean of the coupling strengths for a given overlap η_{45} using ideal TE_3 and TE_4 waveguide modes. (a) Maximally achievable geometric mean of the coupling strengths for a given overlap. For comparison the previous result for coupling of TE_0 and TE_1 modes is shown in light red. (b) The optimal values of the position and thickness of the active material to achieve the solution in (a). The result of the optimization places the material at the bottom of the waveguide for all thicknesses. By symmetry, an equivalent solution exists at the top (position not shown for clarity). (c) The resulting individual coupling strengths for the TE_3 and TE_4 modes for the solution in (a). $g_{\rm rel} = 1$ corresponds to the coupling strength in a completely filled waveguide.

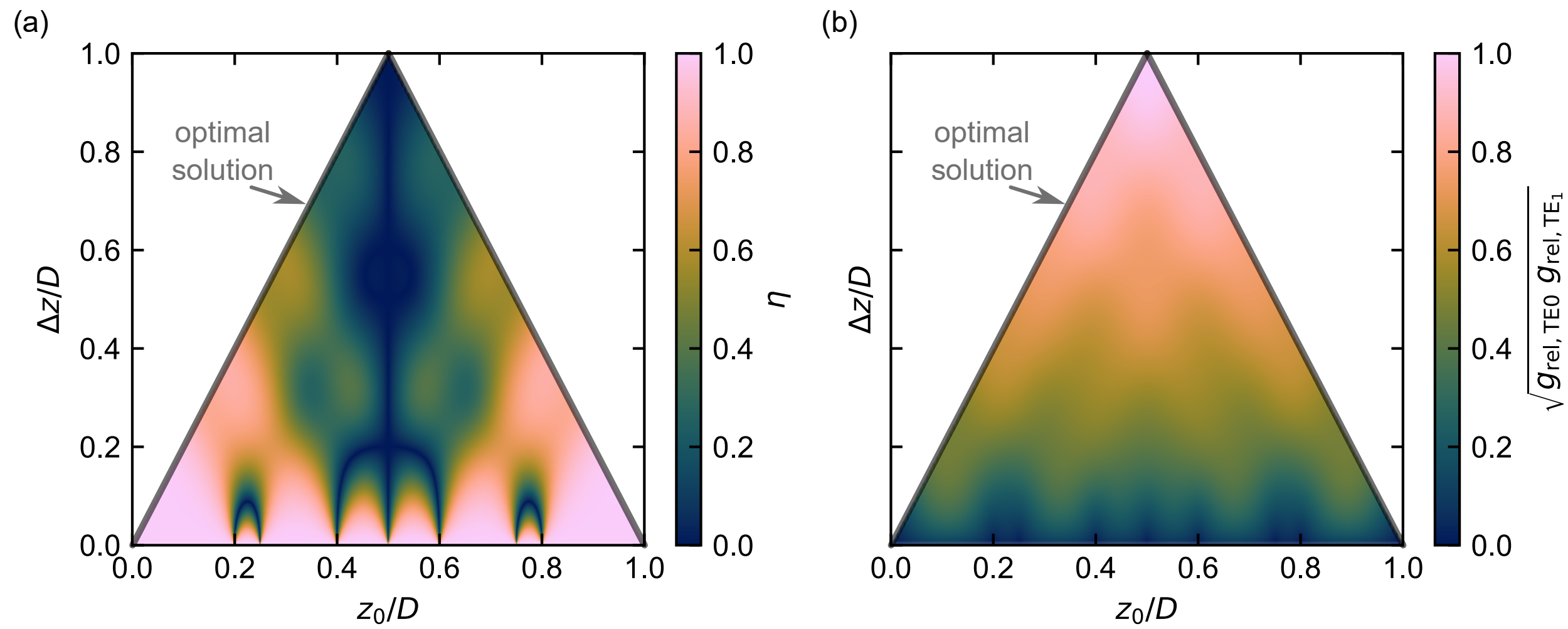

Figure S16: Dependence of (a) the overlap η_{45} and (b) the geometric mean of the coupling strengths on the material thickness Δz and position z_0 for ideal TE_3 and TE_4 waveguide modes. This library is used for the brute-force optimization shown in Figure S15. The optimal solution, corresponding to placement of the active material at the top- or bottommost position of the waveguide, is highlighted by the gray line.

We note that this theoretical treatment of overlap and coupling strength applies both to waveguides and cavities as their idealized mode profiles are identical. The main difference lies in the fact that the energy spacing between modes can be made much smaller in waveguides compared to cavities. Therefore, superstrong coupling in cavities is often performed with higher-order modes, which results in a generally lower coupling strength (see comparison in Figure S15(a)).

S7 Interference-based mode switching

To gain physical insight into the interference-based switching scheme of Figure 5, we derive an analytical expression for the wavevector shift of a polariton mode in response to a change in exciton energy. We consider a single photonic mode with energy $E_{\mathrm{ph}}(\beta)$ at a wavevector β, coupled to an exciton at energy E_{ex} with coupling strength g. The system is described by the 2×2 Hamiltonian:

$$H = \begin{pmatrix} E_{\mathrm{ph}}(\beta) & g \\ g & E_{\mathrm{ex}} \end{pmatrix}, \tag{7}$$

with eigenvalues

$$E_j(\beta, E_{\mathrm{ex}}) = \frac{E_{\mathrm{ph}}(\beta) + E_{\mathrm{ex}}}{2} \pm \frac{1}{2}\sqrt{\delta_0^2 + 4g^2}, \tag{8}$$

where the sign is $-$ for $j = 1$ (lower polariton) and $+$ for $j = 2$ (upper polariton), and $\delta_0(\beta) = E_{\mathrm{ph}}(\beta) - E_{\mathrm{ex}}$ is the photon-exciton detuning. The exciton Hopfield coefficients of the two polariton branches are

$$|X_j|^2 = \frac{1}{2}\left(1 \mp \frac{\delta_0}{\sqrt{\delta_0^2 + 4g^2}}\right), \qquad |C_j|^2 = 1 - |X_j|^2, \tag{9}$$

where the sign is $-$ for $j = 1$ and $+$ for $j = 2$.

In the switching scheme of Figure 5, a laser at fixed energy E_L excites a polariton at wavevector β determined by the resonance condition $E_j(\beta, E_{\mathrm{ex}}) = E_L$. To find how β responds to a shift in E_{ex} at constant E_L, we apply implicit differentiation:

$$\frac{d\beta}{dE_{\mathrm{ex}}} = -\frac{\partial E_j / \partial E_{\mathrm{ex}}}{\partial E_j / \partial \beta}. \tag{10}$$

Evaluating the partial derivatives of Equation (8) yields:

$$\frac{\partial E_j}{\partial E_{\text{ex}}} = \frac{1}{2}\left(1 \mp \frac{\delta_0}{\sqrt{\delta_0^2 + 4g^2}}\right) = |X_j|^2, \tag{11}$$

$$\frac{\partial E_j}{\partial \beta} = \frac{\partial E_{\text{ph}}}{\partial \beta}\,\frac{1}{2}\left(1 \pm \frac{\delta_0}{\sqrt{\delta_0^2 + 4g^2}}\right) = \hbar v_g^{\text{ph}}\,|C_j|^2, \tag{12}$$

where $v_g^{\text{ph}} = (1/\hbar)\,\partial E_{\text{ph}}/\partial \beta$ is the group velocity of the uncoupled photonic mode. Equation (12) shows that the polariton group velocity is $v_{g,j} = v_g^{\text{ph}}\,|C_j|^2$, i.e. the photon group velocity weighted by the photon fraction. Combining Eqs. (11) and (12) gives the central result:

$$\frac{d\beta}{dE_{\text{ex}}} = -\frac{|X_j|^2}{\hbar\, v_{g,j}} = -\frac{|X_j|^2}{|C_j|^2}\,\frac{1}{\hbar\, v_g^{\text{ph}}}. \tag{13}$$

This expression has the following physical interpretation: polariton modes with a higher exciton fraction $|X_j|^2$ are more sensitive to changes in the exciton energy, while modes with a higher group velocity $v_{g,j}$ are less sensitive because a given energy shift translates into a smaller wavevector change along a steeper dispersion.

In the context of Figure 5, the two co-propagating polaritons originate from different photonic modes (TE_0 and TE_1), each described by an independent 2×2 Hamiltonian with its own photon dispersion, coupling strength, and Hopfield coefficients. Applying Equation (13) to each branch, the figure of merit for the interference-based switching is thus:

$$\text{FOM} = \left|\frac{d\beta_1}{dE_{\text{ex}}} - \frac{d\beta_2}{dE_{\text{ex}}}\right| = \left|\frac{|X_1|^2}{\hbar\, v_{g,1}} - \frac{|X_2|^2}{\hbar\, v_{g,2}}\right|, \tag{14}$$

where the subscripts 1 and 2 refer to the two polariton modes. This FOM is maximized when the two polariton modes have strongly differing ratios of exciton fraction to group velocity, which occurs near the exciton resonance where the Hopfield coefficients change rapidly with the exciton energy (Figure S17).

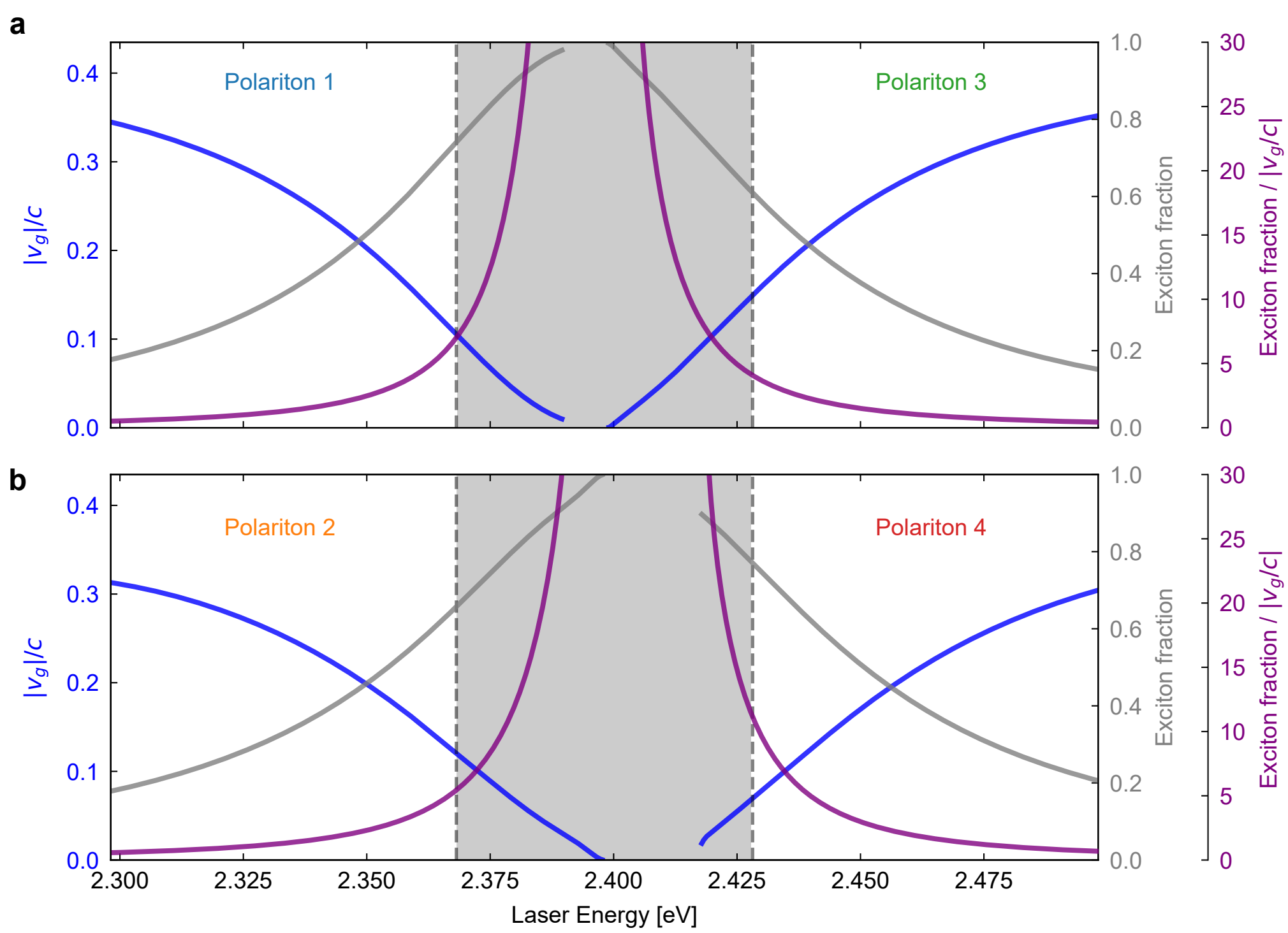

Figure S17: Parameters for the simplified 2×2 model for interference-based mode switching, using the dispersion calculated with Equation (2) of the main text for the low-thickness waveguide (complementing Figure 5). Exciton fraction (gray), group velocity (blue), and their ratio (purple) are shown for (a) Polaritons 1 (left) and 3 (right), and (b) Polaritons 2 (left) and 4 (right). The gray shaded area marks an energy range of $2\gamma_{\mathrm{ex}}$ centered at the exciton, where the system is too lossy for operation.

References

(1) Adl, H. P. et al. Tunable Room-Temperature Polaritons in the Very Strong Coupling Regime in Quasi-2D Ruddlesden–Popper Perovskites. *Advanced Optical Materials* **2025**, *13*, e01392.

(2) Mandal, A.; Xu, D.; Mahajan, A.; Lee, J.; Delor, M.; Reichman, D. R. Microscopic Theory of Multimode Polariton Dispersion in Multilayered Materials. *Nano Letters* **2023**, *23*, 4082–4089.

(3) Georgiou, K.; McGhee, K. E.; Jayaprakash, R.; Lidzey, D. G. Observation of Photon-Mode Decoupling in a Strongly Coupled Multimode Microcavity. *The Journal of Chemical Physics* **2021**, *154*, 124309.

(4) Godsi, M.; Golombek, A.; Balasubrahmaniyam, M.; Schwartz, T. Exploring the Nature of High-Order Cavity Polaritons under the Coupling–Decoupling Transition. *The Journal of Chemical Physics* **2023**, *159*, 134307.

(5) Johnson, A. Observation of Collective Superstrong Coupling of Cold Atoms to a 30-m Long Optical Resonator. *Physical Review Letters* **2019**, *123*.

(6) Tay, F.; Mojibpour, A.; Sanders, S.; Liang, S.; Xu, H.; Gardner, G. C.; Baydin, A.; Manfra, M. J.; Alabastri, A.; Hagenmüller, D.; Kono, J. Multimode Ultrastrong Coupling in Three-Dimensional Photonic-Crystal Cavities. *Nature Communications* **2025**, *16*, 3603.

(7) Mornhinweg, J.; Diebel, L.; Halbhuber, M.; Riepl, J.; Cortese, E.; De Liberato, S.; Bougeard, D.; Huber, R.; Lange, C. Sculpting Ultrastrong Light–Matter Coupling through Spatial Matter Structuring. *Nanophotonics* **2024**, *13*, 1909–1915.

(8) Kuzmin, R.; Mehta, N.; Grabon, N.; Mencia, R.; Manucharyan, V. E. Superstrong Coupling in Circuit Quantum Electrodynamics. *npj Quantum Information* **2019**, *5*, 20.

(9) Han, X.; Zou, C.-L.; Tang, H. X. Multimode Strong Coupling in Superconducting Cavity Piezoelectromechanics. *Physical Review Letters* **2016**, *117*, 123603.

(10) Balasubrahmaniyam, M.; Genet, C.; Schwartz, T. Coupling and Decoupling of Polaritonic States in Multimode Cavities. *Physical Review B* **2021**, *103*, L241407.

(11) Tserkezis, C.; Fernández-Domínguez, A. I.; Gonçalves, P. A. D.; Todisco, F.; Cox, J. D.; Busch, K.; Stenger, N.; Bozhevolnyi, S. I.; Mortensen, N. A.; Wolff, C. On the Applicability of Quantum-Optical Concepts in Strong-Coupling Nanophotonics. *Reports on Progress in Physics* **2020**, *83*, 082401.
